# Pse-in-One: a web server for generating various modes of pseudo components of DNA, RNA, and protein sequences

**DOI:** 10.1093/nar/gkv458

**Published:** 2015-05-09

**Authors:** Bin Liu, Fule Liu, Xiaolong Wang, Junjie Chen, Longyun Fang, Kuo-Chen Chou

**Affiliations:** 1 School of Computer Science and Technology, Harbin Institute of Technology Shenzhen Graduate School, Shenzhen, Guangdong 518055, China; 2 Key Laboratory of Network Oriented Intelligent Computation, Harbin Institute of Technology Shenzhen Graduate School, Shenzhen, Guangdong 518055, China; 3 Gordon Life Science Institute, Belmont, MA 02478, USA; 4 Center of Excellence in Genomic Medicine Research (CEGMR), King Abdulaziz University, Jeddah 21589, Saudi Arabia

## Abstract

With the avalanche of biological sequences generated in the post-genomic age, one of the most challenging problems in computational biology is how to effectively formulate the sequence of a biological sample (such as DNA, RNA or protein) with a discrete model or a vector that can effectively reflect its sequence pattern information or capture its key features concerned. Although several web servers and stand-alone tools were developed to address this problem, all these tools, however, can only handle one type of samples. Furthermore, the number of their built-in properties is limited, and hence it is often difficult for users to formulate the biological sequences according to their desired features or properties. In this article, with a much larger number of built-in properties, we are to propose a much more flexible web server called Pse-in-One (http://bioinformatics.hitsz.edu.cn/Pse-in-One/), which can, through its 28 different modes, generate nearly all the possible feature vectors for DNA, RNA and protein sequences. Particularly, it can also generate those feature vectors with the properties defined by users themselves. These feature vectors can be easily combined with machine-learning algorithms to develop computational predictors and analysis methods for various tasks in bioinformatics and system biology. It is anticipated that the Pse-in-One web server will become a very useful tool in computational proteomics, genomics, as well as biological sequence analysis. Moreover, to maximize users’ convenience, its stand-alone version can also be downloaded from http://bioinformatics.hitsz.edu.cn/Pse-in-One/download/, and directly run on Windows, Linux, Unix and Mac OS.

## INTRODUCTION

To expedite analyses of increasing number of biological sequences, many machine-learning algorithms have been introduced into computational biology. However, nearly all the existing algorithms can only handle vectors but not sequence samples, as elaborated in (1).

However, a vector defined in a discrete model may completely lose the sequence-order information. To cope with such a dilemma, the idea of pseudo amino acid composition or PseAAC (2,3) was proposed. In addition to the well-known amino acid composition (AAC), PseAAC contains special terms called ‘pseudo components’. It is through these terms that the sequence order effects are approximately reflected (2,3).

Ever since it was introduced in 2001, the concept of PseAAC has rapidly penetrated into almost all the areas of computational proteomics (see a long list of references cited in a recent paper (4)).

Encouraged by the successes of using PseAAC to deal with protein/peptide sequences, the corresponding approaches were proposed recently to deal with DNA sequences (5–7) and RNA sequences (8).

Because this kind of approaches have been widely and increasingly used in many areas of computational biology, a number of web servers and stand-alone programs were developed for generating varieties of pseudo components for DNA sequences (9,10), RNA sequences (8) and protein sequences (4,11–13).

The aforementioned web servers did indeed play important roles in stimulating the development of computational biology, however, they have the following problems: (i) lack of flexibility, i.e. they can each only handle one type of biological sequences (DNA, RNA or protein); (ii) un-catching up, i.e. they have missed some pseudo component modes proposed very recently; (iii) limitation, i.e. they cannot cover all the possible physicochemical properties, nor those defined by users themselves.

The present study was initiated in an attempt to overcome the three shortcomings by establishing a new and much more powerful web server.

## MATERIALS AND METHODS

Given a DNA/RNA/protein sequence **S** as expressed by

(1) \begin{equation*} {\bf S} = {\rm R}_1 {\rm R}_2 {\rm R}_3 {\rm R}_4 {\rm R}_5 {\rm R}_6 {\rm R}_7 \cdots {\rm R}_L \end{equation*}

where R_1_ denotes the first residue of **S**, R_2_ the second one, and so forth; *L* is the length of the sequence. In order to deal with it by means of the existing machine-learning algorithms such as SVM (support vector machine) and NN (neural network), the sequence must be first converted into a dimension-fixed vector containing its key features, the so-called feature vector. However, this is by no means an easy job because different biological sequences may have different lengths with a huge number of possible sequence patterns.

Here, we are to propose a powerful web server, called Pse-in-One, by which users can generate all the possible pseudo components for DNA, RNA and protein sequences. It covers a total of 28 different modes, of which 14 for DNA sequences (5–7,9–10,14–17), six for RNA sequences (8,18) and eight for protein sequences (2–3,16,19–21).

**Figure 1.**
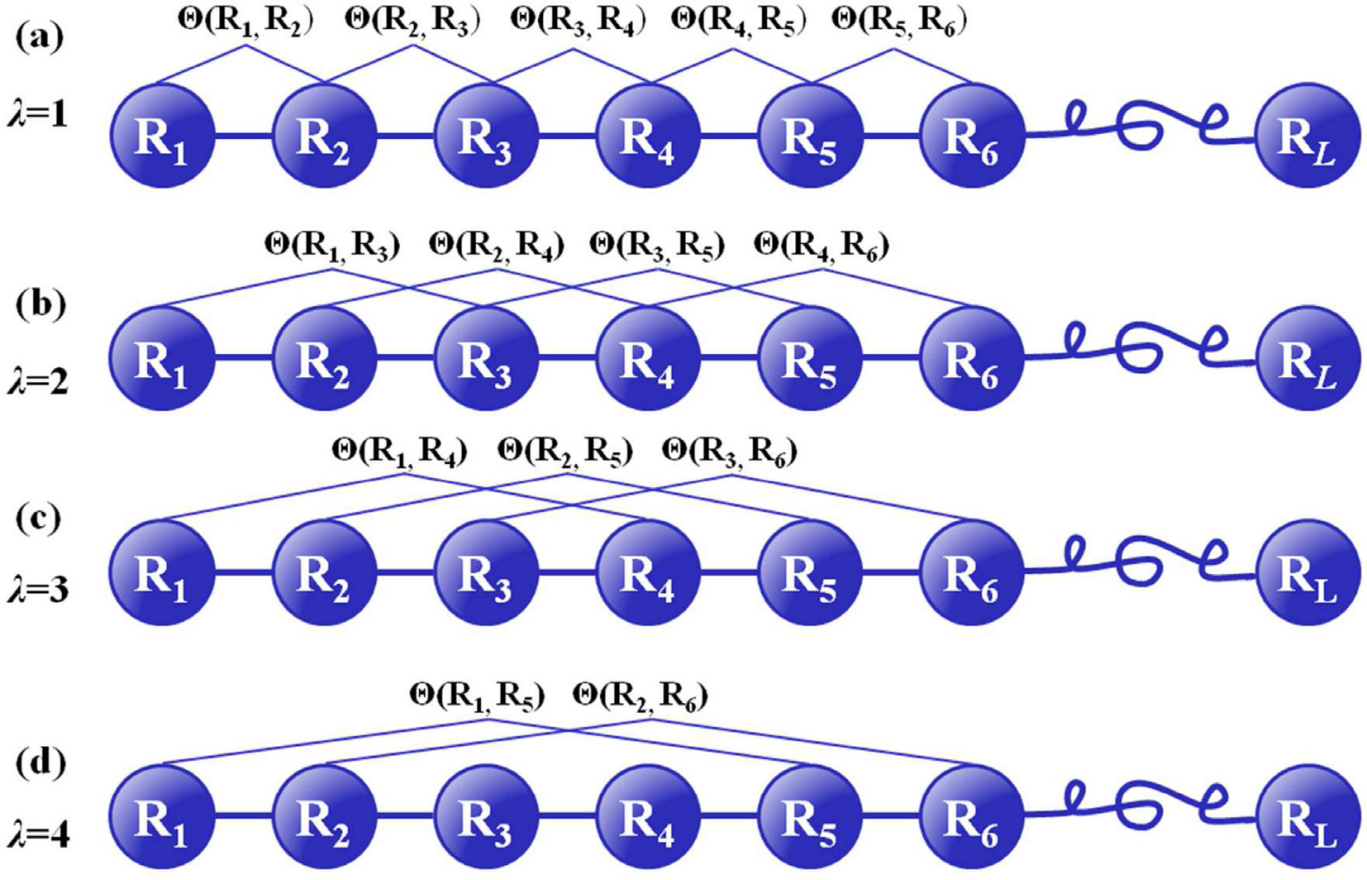
A schematic drawing to show the sequence-order correlation along a DNA/RNA/protein sequence of Eq. (1) for (**a**) the coupling between all the contiguous residues, (**b**) that between the second most contiguous residues, (**c**) that between all the third most contiguous residues and (**d**) that between all the fourth most contiguous residues, where Ξ(*R_i_, R_j_*) is a coupling factor between *i*th and *j*th residues, which is a function defined by users in terms of their physicochemical properties. See (10,27) for more details about this.

Pse-in-One contains three sub web servers: (1) PseDAC-General, (2) PseRAC-General and (3) PseAAC-General. Each of them contains three categories. The first one is to generate the pseudo components for the short-range or local sequence order information by counting the occurrence frequencies of the *k* nearest residues along the sequence **S**. The second and third categories are to generate the pseudo components for the long-range or global sequence order information by counting, respectively, the auto and special correlations of residues along the sequence as shown in Figure 1. Because of space limit, given below is only a brief introduction; for more details about the three sub web servers, see Supplementary Description S1.

**Table 1.** List of the 14 vector modes that can be generated by PseDAC-General for DNA sequences

Category	Mode
1. Nucleic acid composition	(1) Basic kmer (Kmer) (15)
	(2) Reverse complementary kmer (RevKmer) (17,22)
2. Autocorrelation	(3) Dinucleotide-based auto covariance (DAC) (16)
	(4) Dinucleotide-based cross covariance (DCC) (16)
	(5) Dinucleotide-based auto-cross covariance (DACC) (16)
	(6) Trinucleotide-based auto covariance (TAC) (16)
	(7) Trinucleotide-based cross covariance (TCC) (16)
	(8) Trinucleotide-based auto-cross covariance (TACC) (16)
3. Pseudo nucleotide composition	(9) Pseudo dinucleotide composition (PseDNC) (5)
	(10) Pseudo *k*-tuple nucleotide composition (PseKNC) (6,7)
	(11) General parallel correlation pseudo dinucleotide composition (PC-PseDNC-General) (9,10)
	(12) General parallel correlation pseudo trinucleotide composition (PC-PseTNC-General) (9,10)
	(13) General series correlation pseudo dinucleotide composition (SC-PseDNC-General) (9,10)
	(14) General series correlation pseudo trinucleotide composition (SC-PseTNC-General) (9,10)

PseDAC-General is the abbreviation for ‘pseudo deoxyribonucleic acid compositions for DNA sequences’. It contains 14 different modes to generate various feature vectors for DNA sequences, and can be grouped into the following three categories (Table 1).

The first category is of nucleic acid composition that contains two modes, basic Kmer (Kmer) (15) and reverse complementary kmer (RevKmer) (17,22). Kmer means the subsequence of a DNA sequence containing *k* neighboring nucleic acids. The reverse complementary kmer is a variant of the kmer, in which the kmers are not expected to be strand-specific, so reverse complements are collapsed into a single feature. Therefore, both the Kmer and RevKmer can represent the local DNA sequence composition.

The second category is of autocorrelation that contains six modes, reflecting different correlation manners between two dinucleotides or trinucleotides along a DNA sequence via their physicochemical properties. Of the six modes, three (DAC, DCC and DACC) are based on the 148 physicochemical indices of dinucleotides extracted from (8,14); and the other three (TAC, TCC and TACC) based on the 12 physicochemical indices of trinucleotides extracted from (8).

The third category is of pseudo nucleotide composition that contains six modes by incorporating the global or long-range sequence order information into the feature vectors via the physicochemical properties of dinucleotides or trinucleotides. Of the six modes, PseDNC is based on the six local DNA structural properties of dinucleotides; PseKNC extends the PseDNC to the level that can incorporate *k*-tuple nucleotides as well; PC-PseDNC-General and SC-PseDNC-General are two general modes based on the properties of dinucleotides, by which users can generate parallel correlation components and series correlation components, via not only selecting the properties from the 148 built-in indices but also uploading the properties defined by themselves; PC-PseTNC-General and SC-PseTNC-General are another two general modes but based on the properties of trinucleotides with 12 built-in indices, by which users can do the same as in PC-PseDNC-General and SC-PseDNC-General, respectively.

**Table 2.** List of six vector modes that can be generated by PseRAC-General for RNA sequences

Category	Mode
Nucleic acid composition	(1) Basic kmer (Kmer) (18)
Autocorrelation	(2) Dinucleotide-based auto covariance (DAC) (16,21)
	(3) Dinucleotide-based cross covariance (DCC) (16,21)
	(4) Dinucleotide-based auto-cross covariance (DACC) (16,21)
Pseudo nucleotide composition	(5) General parallel correlation pseudo dinucleotide composition (PC-PseDNC-General) (8)
	(6) General series correlation pseudo dinucleotide composition (SC-PseDNC-General) (8)

PseRAC-General is the abbreviation for ‘pseudo ribonucleic acid compositions for RNA sequences’. It contains six different modes to generate various feature vectors for RNA sequences, and can be grouped into the following three categories (Table 2).

The first category is of basic kmer, where the occurrence frequencies of *k* neighboring nucleic acids (kmers) are used to reflect the short-range or local sequence compositions of RNA.

The second category is of autocorrelation that contains three modes, reflecting the level of correlation between two dinucleotides along a RNA sequence in terms of their physicochemical properties. Of the three modes, one is of DAC, one is of DCC, and one is of DACC that combines DAC and DCC. Users can use each of these modes to generate their desired RNA feature vectors by selecting 22 built-in properties from (8,14), and the properties defined by their own.

The third category is of pseudo nucleotide composition that contains two modes: PC-PseDNC-General and SC-PseDNC-General. The former can generate the parallel correlation (8) components for RNA sequences via the properties selected from 22 built-in physiochemical indices from (8,14) or the user-defined properties, while the latter can generate the corresponding series correlation (8) components via the same manner.

**Table 3.** List of eight vector modes that can be generated by PseAAC-General for protein sequences

Category	Mode
Amino acid composition	(1) Basic kmer (Kmer) (20)
Autocorrelation	(2) Auto covariance (AC) (16,21)
	(3) Cross covariance (CC) (16,21)
	(4) Auto-cross covariance (ACC) (16,21)
Pseudo amino acid composition	(5) Parallel correlation pseudo amino acid composition (PC-PseAAC) (2)
	(6) Series correlation pseudo amino acid composition (SC-PseAAC) (3)
	(7) General parallel correlation pseudo amino acid composition (PC-PseAAC-General) (2,4)
	(8) General series correlation pseudo amino acid composition (SC-PseAAC-General)) (3,4)

PseAAC-General is the abbreviation for ‘pseudo amino acid composition for protein sequences’. It contains eight different modes to generate various feature vectors for protein sequences, and can be grouped into the following three categories (Table 3).

The first category is of basic kmer, where the occurrence frequencies of *k* neighboring amino acids (kmers) are used to reflect the short-range or local sequence compositions of protein.

The second category is of autocorrelation that contains three modes, reflecting three different manners in counting the correlations along a protein chain via the 547 amino acid physicochemical properties extracted from AAindex (19). Of the three, the first one is of auto covariance (AC) that incorporates the correlation of the same property between two amino acids; the second one is of cross covariance (CC) that incorporates the correlation of the different properties between two amino acids; and the third one is of auto-cross covariance (ACC) that is a combination of AC and CC. Besides, the three modes also have the function to generate the feature vectors by user-defined properties.

The third category is of pseudo amino acid composition for incorporating the global or long-range sequence order information of protein sequences into their feature vectors via the physicochemical properties of their constituent amino acids. It contains four modes: PC-PseAAC, SC-PseAAC, PC-PseAAC-General, and SC-PseAAC-General, where the first and second modes are generating the protein feature vectors by combining the amino acid composition and global sequence-order effects via parallel correlation (2) and series correlation (3) respectively; while the third and fourh modes are the general forms of PC-PseAAC and SC-PseAAC, meaning that, besides the aforementioned 547 physicochemical properties, they also allow to incorporate higher level information such as functional domain (FunD), gene ontology (GO), and sequential evolution (4,23), as well as any user-defined properties.

Accordingly, with Pse-in-One, what users need to do is just to input DNA, RNA, or protein sequences along with their selected parameters. After clicking the Submit button, they can immediately obtain the desired feature vectors ready for most existing machine-learning algorithms to conduct varieties of analyses. Particularly, the feature vectors thus obtained can also be visualized via an intuitive graph called the ‘heat map’ shown on the screen, which is very useful for users to adjust their selected parameters. To our best knowledge, Pse-in-One is so far the first ever web server that can generate all the possible pseudo components for DNA, RNA, and protein sequences, as well as those even with the properties defined by users themselves. Therefore, it is very flexible with extremely high capacity and wide coverage, allowing users to have many choices to generate their desired pseudo components for in-depth analyzing varieties of DNA, RNA or protein/peptide sequences.

## WEB SERVER

### Input

**Figure 2.**
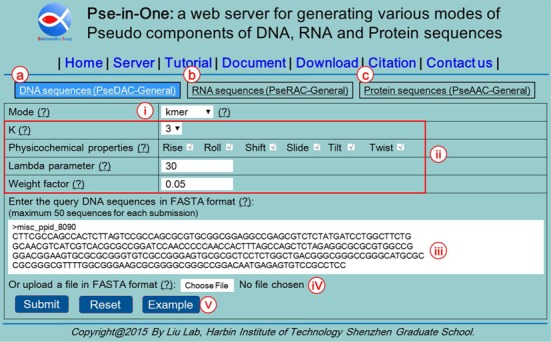
A semi screenshot to show that Pse-in-One contains three sub-servers. They are: (**a**) PseDAC-General, (**b**) PseRAC-General and (**c**) PseAAC-General. For each of the three sub-servers, users can generate their desired feature vectors via the buttons marked with (i), (ii), (iii), (iv) and (v), respectively.

For all the three sub web servers, the input is a set of DNA, RNA or protein sequences in FASTA format, which can be either uploaded as a single file or copied/pasted into the input box. The total number of input sequences is limited to 50 for each submission. There is no such limitation for the downloadable stand-alone program of Pse-in-One. For each of the 28 modes, the sample input sequences, and parameters can be automatically set by clicking the Example button so as to help the users to understand the input data format, and test the Pse-in-One. Shown in Figure 2 is the input page of Pse-in-One.

For the PseDAC-General, 14 modes of DNA sequences can be computed. For Kmer and RevKmer, the only parameter to be chosen by the users is *k*, the length of kmers, which can be set as any integer ranging from 1 to 6. For DAC, DCC, DACC, TAC, TCC and TACC, the users can specify the value for *lag*, select physicochemical properties from the built-in indices, and upload the user-defined indices as a single file. For PseDNC, PseKNC, PC-PseDNC-General, PC-PseTNC-General, SC-PseDNC-General, SC-PseTNC-General, the users can specify values for *lambda* and *weight*. Comprehensive built-in and user-defined physicochemical properties can be selected to generate these feature vectors.

For the PseRAC-General, six modes of RNA sequences can be computed. For Kmer, the users can specify value for *k* as any integer ranging from 1 to 6. For DAC, DCC and DACC, the users should set the value for *lag*, and the physicochemical properties either from the built-in indices or the user-defined indices. For PC-PseDNC-General and SC-PseDNC-General, the values of two parameters *lambda* and *weight* as well as the physicochemical properties should be specified by the users.

For the PseAAC-General, eight modes of protein sequences can be computed. For Kmer, the value of parameter *k* should be set by the users. The dimension of the Kmer feature vector increases rapidly with the values of *k*; for example, when *k* is set at 3, the dimension of the corresponding feature vector is 8000. Therefore, Kmer only allows the users to set the values of *k* at 1, 2 and 3. For larger *k* values, the users should use the stand-alone program to compute. For AC, CC and ACC, the users should set the value of *lag* and select the physicochemical properties of amino acids from the built-in and user-defined indices. For PC-PseAAC, SC-PseAAC, PC-PseAAC-General and SC-PseAAC-General, the values of two parameters *lambda* and *weight* as well as the physicochemical properties should be set by the users.

### Output

**Figure 3.**
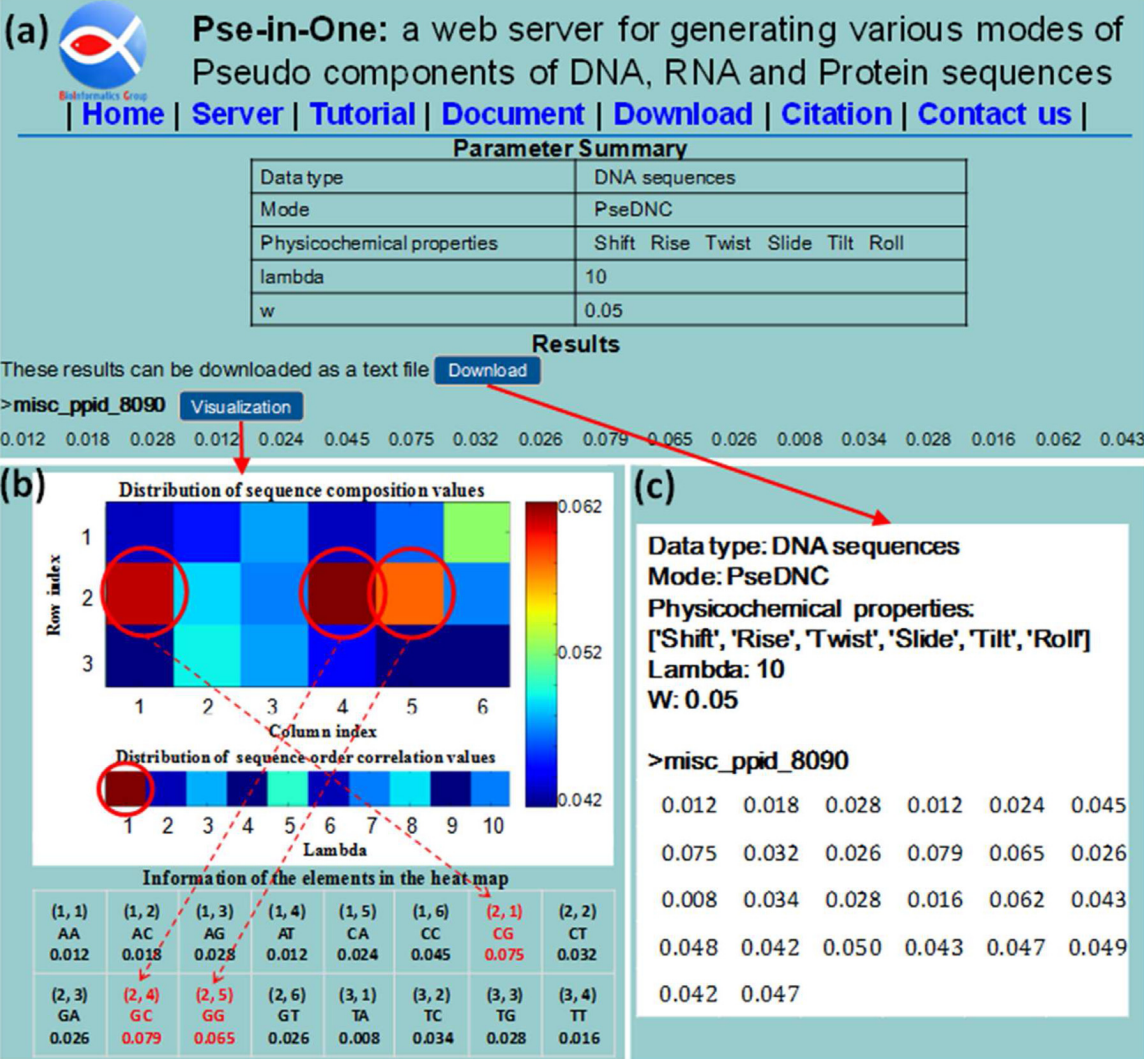
A semi screenshot to show the output from Pse-in-One. It contains three panels: (**a**) a summary of the input sequence type, selected feature vector mode, parameter and the resultant feature vector; (**b**) a heat-map for visualization with the horizontal and vertical axes for the feature vector's column and row indexes, respectively; (**c**) an example of output ready for downstream computational analyses. See the main texts for more explanation.

Shown in Figure 3 is a display of the computed results when using the PseDNC mode in the sub web server PseDAC-General and using the provided example data (DNA sequence misc_ppid_8090 with *λ* = 10 and *w* = 0.05) as the input. The figure contains three panels: (a) a summary of the input sequence type, selected feature vector mode and parameter, and the resultant feature vector; (b) a heat-map for visualization with the horizontal and vertical axes for the feature vector's column and row indexes, respectively; (c) an example of output ready for downstream computational analyses.

Figure 3a contains two parts: the first part is a 2 × 5 table listing the selected mode, values of parameters, physicochemical properties, etc.; the second part shows the feature vectors derived from the input sequences.

Figure 3b shows the characters of the resultant feature vectors via an intuitive graphical representation called ‘heat map’, where the upper heat map visualizes the occurrence frequencies of the local sequence composition (kmers), and the lower one visualizes the sequence-order correlation captured by PseDNC. The feature names and the corresponding values associated with the upper heat map are given in a 2 × 8 table located at the bottom of the panel according to their column and row indexes. Three kmers (GC, CG and GG) marked with the red cycles have the top three values, indicating that the nucleic acid residues C, G and their combinations are abundant in this DNA sequence. As we can see from the lower heat map, the feature with *λ* = 1 has the top value, indicating the importance of the neighboring kmers (cf. Figure 1a) for such a sequence. This kind of intuitive heat map can help users to select their desired parameters and modes according to the purposes of their studies.

Figure 3c shows the feature vectors thus obtained that can be directly downloaded as a separate file. For the stand-alone program, the output files support the following three formats: the LIBSVM format, the CSV format of Excel, and the tab-delimited format that can be directly imported into MATLAB. All these files are suitable for downstream computational analyses, such as machine learning.

### Applications of Pse-in-One

According to the Chou's 5-step rule (23) and carried out in a series of recent publications (see, e.g. (5,7,24–26)), to establish a really useful statistical predictor for a biological system, one needs to consider the following five guidelines: (i) construct or select a valid benchmark dataset to train and test the predictor; (ii) formulate the statistical samples with an effective mathematical expression that can truly reflect their intrinsic correlation with the target to be predicted; (iii) introduce or develop a powerful machine learning algorithm (or engine) to operate the prediction; (iv) properly perform cross-validation tests to objectively evaluate the anticipated accuracy of the model; (v) establish a user-friendly web-server for the predictor that is accessible to the public.

In the aforementioned 5-step rule, the most difficult and time-consuming task is in the second step; i.e. how to find an effective digit feature vector to represent the biological sequence concerned. There are many ongoing studies focused on different topics and targets, and hence many different modes of feature vectors are needed to deal with them, as reported in more than hundred papers cited in Supplementary Description S2. Fortunately, using the Pse-in-One web server, we can easily generate all these desired feature vectors by just selecting different parameters. Accordingly, its practical application value is self-evident.

For example, in the topic of identifying recombination spots (5), the authors used the feature vectors of pseudo dinucleotide composition (PseDNC) to represent the DNA samples after many mathematical derivations. In contrast, if using the current web server, we can immediately obtain exactly the same feature vectors by selecting ‘PseDNC’ for the mode and setting the parameters at *λ* = 3 and *w* = 0.05. The output thus generated can be downloaded into a text file accepted by many machine-learning algorithms for further analysis, significantly speeding up the process.

## DISCUSSION

Compared with the existing web servers and stand-alone tools in converting biological sequences into various feature vectors, Pse-in-One has the following advantages. (i) It is so far the first web server that can generate all the existing pseudo components for DNA, RNA and protein sequences that might be acquired by separately trying many different web servers and stand-alone tools such as PseAAC (11), PseAAC-Builder (12), propy (13), PseAAC-General (4), PseKNC (9,10) and PseKNC-General (8). (ii) As mentioned above, it contains 148, 22 and 547 built-in physicochemical properties for users to select in generating feature vectors for DNA, RNA and protein sequences, respectively. Accordingly, the total possible different feature vectors generated by Pse-in-One for a DNA sequence would be 3.57 × 10^44^, that for an RNA sequence would be 4.19 × 10^6^, and that for a protein sequence would be 4.61 × 10^164^, meaning large enough to nearly cover all the possible cases. (iii) Furthermore, it also allows users to generate those pseudo components according to the properties defined by users’ own, which is beyond the reach of any existing web server in this area.

## AVAILABILITY

http://bioinformatics.hitsz.edu.cn/Pse-in-One/

## SUPPLEMENTARY DATA

Supplementary Data are available at NAR Online.

SUPPLEMENTARY DATA
