# Supplementary material for: Pse-in-One: a web server for generating various modes of pseudo components of DNA, RNA, and protein sequences
Source: Nucleic Acids Res. 2015 May 9;43(Web Server issue):W65–71. doi: 10.1093/nar/gkv458 (PMC4489303; doi:10.1093/nar/gkv458)
Supplement: SUPPLEMENTARY DATA [file supp_43_W1_W65__index.html]

Pse-in-One: a web server for generating various modes of pseudo components of DNA, RNA, and protein sequences — SUPPLEMENTARY DATA 

# Pse-in-One: a web server for generating various modes of pseudo components of DNA, RNA, and protein sequences

## SUPPLEMENTARY DATA

- SUPPLEMENTARY DATA
- SUPPLEMENTARY DATA
