# Supplementary material for: Pse-in-One: a web server for generating various modes of pseudo components of DNA, RNA, and protein sequences
Source: Nucleic Acids Res. 2015 May 9;43(Web Server issue):W65–71. doi: 10.1093/nar/gkv458 (PMC4489303; doi:10.1093/nar/gkv458)
Supplement: SUPPLEMENTARY DATA [file supp_gkv458_nar-00118-web-b-2015-File002.pdf]

Pse-in-One: a web server for generating various modes of pseudo components of DNA, RNA, and protein sequences

2015-04-08

Home-page: http://bioinformatics.hitsz.edu.cn/Pse-in-One/

Content

1. DNA....3
1.1 Deoxyribonucleic acid composition 3
1.1.1 Basic kmer (Kmer)....3
1.1.2 Reverse complementary kmer (RevKmer)....3
1.2 Autocorrelation 3
1.2.1 Dinucleotide-based auto covariance (DAC)....3
1.2.2 Dinucleotide-based cross covariance (DCC) 4
1.2.3 Dinucleotide-based auto-cross covariance (DACC) 4
1.2.4 Trinucleotide-based auto covariance (TAC)....4
1.2.5 Trinucleotide-based cross covariance (TCC) 5
1.2.6 Trinucleotide-based auto-cross covariance (TACC) 5
1.3 Pseudo deoxyribonucleic acid composition....5
1.3.1 Pseudo dinucleotide composition (PseDNC) 5
1.3.2 Pseudo k-tuple nucleotide composition (PseKNC)....6
1.3.3 General parallel correlation pseudo dinucleotide composition (PC-PseDNC-General)...7
1.3.4 General parallel correlation pseudo trinucleotide composition (PC-PseTNC-General)...8
1.3.5 General series correlation pseudo dinucleotide composition (SC-PseDNC-General)......9
1.3.6 General series correlation pseudo trinucleotide composition (SC-PseTNC-General) ...10
2. RNA.... 11
2.1 Ribonucleic acid composition.... 11
2.1.1 Basic kmer (Kmer)....11
2.2 Autocorrelation 11
2.2.1 Dinucleotide-based auto covariance (DAC)....11
2.2.2 Dinucleotide-based cross covariance (DCC) 12
2.2.3 Dinucleotide-based auto-cross covariance (DACC) 12
2.3 Pseudo ribonucleic acid composition.... 12
2.3.1 General parallel correlation pseudo dinucleotide composition (PC-PseDNC-General).13
2.3.2 General series correlation pseudo dinucleotide composition (SC-PseDNC-General)....13
3. Protein.... 14
3.1 Amino acid composition 14
3.1.1 Basic kmer (Kmer)....15
3.2 Autocorrelation 15
3.2.1 Auto covariance (AC) 15
3.2.2 Cross covariance (CC) 15
3.2.3 Auto-cross covariance (ACC) 16
3.3 Pseudo amino acid composition.... 16
3.3.1 Parallel correlation pseudo amino acid composition (PC-PseAAC)....16
3.3.2 Series correlation pseudo amino acid composition (SC-PseAAC) 17
3.3.3 General parallel correlation pseudo amino acid composition (PC-PseAAC-General)...19
3.3.4 General series correlation pseudo amino acid composition (SC-PseAAC-General)......20
Table 1. The names of the 148 physicochemical indices for dinucleotides (DNA)..... 22
Table 2. The names of the 12 physicochemical indices for trinucleotides (DNA). 23
Table 3. The names of the 6 physicochemical indices for dinucleotides (DNA)......... 23
Table 4. The names of the 22 physicochemical indices for dinucleotides (RNA)....... 23
Table 5. The names of the 547 physicochemical indices for amino acids. 24
Table 6. The names of the 3 physicochemical indices for amino acids. 27
Table 7. The names of the 2 physicochemical indices for amino acids. 28
References.... 28

1. DNA

1.1 Deoxyribonucleic acid composition

1.1.1 Basic kmer (Kmer)

Basic kmer (1) is the simplest approach to represent the DNAs, in which the DNA sequences are represented as the occurrence frequencies of *k* neighboring nucleic acids. This approach has been successfully applied to human gene regulatory sequence prediction (2,3), enhancer identification (1), etc.

1.1.2 Reverse complementary kmer (RevKmer)

The reverse complementary kmer (2,3) is a variant of the basic kmer, in which the kmers are not expected to be strand-specific, so reverse complements are collapsed into a single feature. For example, if *k*=2, there are totally 16 basic kmers ('AA', 'AC', 'AG', 'AT', 'CA', 'CC', 'CG', 'CT', 'GA', 'GC', 'GG', 'GT', 'TA', 'TC', 'TG', 'TT'), but by removing the reverse complementary kmers, there are only 10 distinct kmers in the reverse complementary kmer approach ('AA', 'AC', 'AG', 'AT', 'CA', 'CC', 'CG', 'GA', 'GC', 'TA'). For more information of this approach, please refer to (2,3).

1.2 Autocorrelation

1.2.1 Dinucleotide-based auto covariance (DAC)

Suppose a DNA sequence **D** with *L* nucleic acid residues; i.e.

$$\mathbf{D} = \mathrm{R}_1\mathrm{R}_2\mathrm{R}_3\mathrm{R}_4\mathrm{R}_5\mathrm{R}_6\mathrm{R}_7 \cdots \mathrm{R}_L \tag{1}$$

where R_1 represents the nucleic acid residue at the sequence position 1, R_2 the nucleic acid residue at position 2 and so forth.

The DAC (4-6) measures the correlation of the same physicochemical index between two dinucleotides separated by a distance of *lag* along the sequence, which can be calculated as:

$$\mathrm{DAC}(u, lag) = \sum_{i=1}^{L-lag-1} (P_u(\mathrm{R}_i\mathrm{R}_{i+1}) - \overline{P}_u)(P_u(\mathrm{R}_{i+lag}\mathrm{R}_{i+lag+1}) - \overline{P}_u) / (L - lag - 1) \tag{2}$$

where *u* is a physicochemical index, *L* is the length of the DNA sequence, $P_u(\mathrm{R}_i\mathrm{R}_{i+1})$ means the numerical value of the physicochemical index *u* for the dinucleotide $\mathrm{R}_i\mathrm{R}_{i+1}$ at position *i*, $\overline{P}_u$ is the average value for physicochemical index *u* along the whole sequence:

$$\overline{P}_u = \sum_{j=1}^{L-1} P_u(\mathrm{R}_j\mathrm{R}_{j+1}) / (L-1) \tag{3}$$

In such a way, the length of DAC feature vector is $N*$LAG, where N is the number of physicochemical indices (**Table 1**) extracted from two papers (6,7), and LAG is the maximum of *lag* (*lag* = 1, 2, … , LAG).

1.2.2 Dinucleotide-based cross covariance (DCC)

Given a DNA sequence **D** (**Eq. 1**), the DCC (4,6) approach measures the correlation of two different physicochemical indices between two dinucleotides separated by *lag* nucleic acids along the sequence, which can be calculated by:

$$\text{DCC}(u_1,u_2,lag) = \sum_{i=1}^{L-lag-1} (P_{u_1}(R_i R_{i+1}) - \overline{P}_{u_1})(P_{u_2}(R_{i+lag} R_{i+lag+1}) - \overline{P}_{u_2}) / (L - lag - 1) \quad (4)$$

where u_1, u_2 are two different physicochemical indices, L is the length of the DNA sequence, $P_{u_1}(\text{R}_i\text{R}_{i+1})$ ($P_{u_2}(\text{R}_i\text{R}_{i+1})$) is the numerical value of the physicochemical index u_1 (u_2) for the dinucleotide R_iR_{i+1} at position i, $\overline{P}_{u_1}$ ($\overline{P}_{u_2}$) is the average value for physicochemical index value u_1(u_2) along the whole sequence:

$$\overline{P_u} = \sum_{j=1}^{L-1} P_u(\text{R}_j \text{R}_{j+1}) / (L-1) \quad (5)$$

In such a way, the length of the DCC feature vector is N*(N-1)*LAG, where LAG is the maximum of *lag* (*lag*=1, 2, …, LAG); N is the number of physicochemical indices (**Table 1**).

1.2.3 Dinucleotide-based auto-cross covariance (DACC)

DACC (4,6) is a combination of DAC and DCC. Therefore, the length of the DACC feature vector is N*N*LAG, where N is the number of physicochemical indices (**Table 1**) and LAG is the maximum of *lag* (*lag* = 1, 2, …, LAG).

1.2.4 Trinucleotide-based auto covariance (TAC)

Given a DNA sequence **D** (**Eq. 1**), the TAC approach (4-6) measures the correlation of the same physicochemical index between two trinucleotides separated by *lag* nucleic acids along the sequence, which can be calculated as:

$$\text{TAC}(lag,u) = \sum_{i=1}^{L-lag-2} (P_u(\text{R}_i \text{R}_{i+1} \text{R}_{i+2}) - \overline{P}_u)(P_u(\text{R}_{i+lag} \text{R}_{i+lag+1} \text{R}_{i+lag+2}) - \overline{P}_u) / (L - lag - 2) \quad (6)$$

where u is a physicochemical index, L is the length of the DNA sequence, $P_u(\text{R}_i\text{R}_{i+1}\text{R}_{i+2})$ represents the numerical value of the physicochemical index u for the trinucleotide $\text{R}_i\text{R}_{i+1}\text{R}_{i+2}$ at position i, $\overline{P}_u$ is the average value for physicochemical index u along the whole sequence:

$$\overline{P_u} = \sum_{j=1}^{L\text{-}2} P_u(\text{R}_j \text{R}_{j+1} \text{R}_{j+2}) / (L-2) \quad (7)$$

In such a way, the length of TAC feature vector is $N*$LAG, where N is the number of physicochemical indices (**Table 2**) extracted from (6), and LAG is the maximum of *lag* (lag=1, 2, …, LAG).

1.2.5 Trinucleotide-based cross covariance (TCC)

Given a DNA sequence **D** (**Eq. 1**), the TCC (4,6) approach measures the correlation of two different physicochemical indices between two trinucleotides separated by *lag* nucleic acids along the sequence, which can be calculated by:

$$\text{TCC}(u_1,u_2,\text{lag})=\sum_{i=1}^{L-lag\text{-}2}(P_{u_1}(\text{R}_i\text{R}_{i+1}\text{R}_{i+2})-\overline{P}_{u_1})(P_{u_2}(\text{R}_{i+lag}\text{R}_{i+lag+1}\text{R}_{i+lag+2})-\overline{P}_{u_2})/(L\text{-}lag\text{-}2) \quad (8)$$

where u_1, u_2 are two physicochemical indices; L is the length of the DNA sequence; $P_{u_1}(\text{R}_i\text{R}_{i+1}\text{R}_{i+2})$ ($P_{u_2}(\text{R}_i\text{R}_{i+1}\text{R}_{i+2})$) represents the numerical value of the physicochemical index u_1 (u_2) for the trinucleotide R_iR_{i+1} R_{i+2} at position i; $\overline{P}_{u_1}$ ($\overline{P}_{u_2}$) is the average value for physicochemical index u_1 (u_2) along the whole sequence:

$$\overline{P}_u=\sum_{j=1}^{L-2}P_u(\text{R}_j\text{R}_{j+1}\text{R}_{j+2})/(L-2) \quad (9)$$

In such a way, the length of TCC feature vector is $N*(N\text{-}1)*$LAG, where N is the number of physicochemical index (**Table 2**) extracted from (6), and LAG is the maximum of *lag* (*lag* = 1, 2, …, LAG).

1.2.6 Trinucleotide-based auto-cross covariance (TACC)

TACC (4,6) is a combination of TAC and TCC. Therefore, the length of the TACC feature vector is $N*N*$LAG, where N is the number of physicochemical indices (**Table 2**) extracted from (6), and LAG is the maximum of *lag* (*lag* = 1, 2, …, LAG).

1.3 Pseudo deoxyribonucleic acid composition

1.3.1 Pseudo dinucleotide composition (PseDNC)

PseDNC (8) is an approach incorporating the contiguous local sequence-order information and the global sequence-order information into the feature vector of the DNA sequence.

Given a DNA sequence **D** (**Eq. 1**), the PseDNC feature vector of **D** is defined:

$$\mathbf{D}=\begin{bmatrix}d_1 & d_2 & \cdots & d_{16} & d_{16+1} & \cdots & d_{16+\lambda}\end{bmatrix}^{\mathbf{T}} \quad (10)$$

where

$$d_k = \begin{cases} \dfrac{f_k}{\sum_{i=1}^{16} f_i + w\sum_{j=1}^{\lambda}\theta_j} & (1 \le k \le 16) \\ \dfrac{w\theta_{k-16}}{\sum_{i=1}^{16} f_i + w\sum_{j=1}^{\lambda}\theta_j} & (17 \le k \le 16+\lambda) \end{cases} \quad (11)$$

where f_k (k=1,2,···,16) is the normalized occurrence frequency of dinucleotides in the DNA sequence; the parameter λ is an integer, representing the highest counted rank (or tier) of the correlation along a DNA sequence; w is the weight factor ranged from 0 to 1; θ_j (j=1,2,···,λ) is called the j-tier correlation factor that reflects the sequence-order correlation between all the most j-tier contiguous dinucleotides along a DNA sequence, which is defined:

$$\begin{cases} \theta_1 = \dfrac{1}{L\text{-}2}\sum_{i=1}^{L-2}\Theta(\mathrm{R}_i\mathrm{R}_{i+1}, \mathrm{R}_{i+1}\mathrm{R}_{i+2}) \\ \theta_2 = \dfrac{1}{L\text{-}3}\sum_{i=1}^{L-3}\Theta(\mathrm{R}_i\mathrm{R}_{i+1}, \mathrm{R}_{i+2}\mathrm{R}_{i+3}) \\ \theta_3 = \dfrac{1}{L\text{-}4}\sum_{i=1}^{L-4}\Theta(\mathrm{R}_i\mathrm{R}_{i+1}, \mathrm{R}_{i+3}\mathrm{R}_{i+4}) & (\lambda < L) \\ \quad\cdots\cdots \\ \theta_\lambda = \dfrac{1}{L\text{-}1\text{-}\lambda}\sum_{i=1}^{L-1-\lambda}\Theta(\mathrm{R}_i\mathrm{R}_{i+1}, \mathrm{R}_{i+\lambda}\mathrm{R}_{i+\lambda+1}) \end{cases} \quad (12)$$

where the correlation function is given by

$$\Theta(\mathrm{R}_i\mathrm{R}_{i+1}, \mathrm{R}_j\mathrm{R}_{j+1}) = \frac{1}{\mu}\sum_{u=1}^{\mu}[P_u(\mathrm{R}_i\mathrm{R}_{i+1}) - P_u(\mathrm{R}_j\mathrm{R}_{j+1})]^2 \quad (13)$$

where μ is the number of physicochemical indices, in this approach, 6 indices reflecting the local DNA structural properties (8) (**Table 3**) are employed to generate the PseDNC feature vector; $P_u(\mathrm{R}_i\mathrm{R}_{i+1})$ ($P_u(\mathrm{R}_j\mathrm{R}_{j+1})$) represents the numerical value of the u-th ($u = 1,2,\cdots,\mu$) physicochemical index of the dinucleotide $\mathrm{R}_i\mathrm{R}_{i+1}$ ($\mathrm{R}_j\mathrm{R}_{j+1}$) at position i (j).

1.3.2 Pseudo *k*-tuple nucleotide composition (PseKNC)

PseKNC (9,10) extends the PseDNC approach by incorporating *k*-tuple nucleotide composition.

Given a DNA sequence **D** (**Eq. 1**), the feature vector of **D** is defined:

$$\mathbf{D} = \begin{bmatrix} d_1 & d_2 & \cdots & d_{4^k} & d_{4^k+1} & \cdots & d_{4^k+\lambda} \end{bmatrix}^{\mathbf{T}} \quad (14)$$

where

$$d_u = \begin{cases} \dfrac{f_u}{\sum_{i=1}^{4^k} f_i + w\sum_{j=1}^{\lambda}\theta_j} & (1 \le u \le 4^k) \\ \dfrac{w\theta_{u-4^k}}{\sum_{i=1}^{4^k} f_i + w\sum_{j=1}^{\lambda}\theta_j} & (4^k \le u \le 4^k + \lambda) \end{cases} \tag{15}$$

where λ is the number of the total counted ranks (or tiers) of the correlations along a DNA sequence; f_u (u=1,2,⋯,4^k) is the frequency of oligonucleotide that is normalized to $\sum_{i=1}^{4^k} f_i = 1$; w is a weight factor; θ_j is given by

$$\theta_j = \frac{1}{L\text{-}j\text{-}1}\sum_{i=1}^{L-j-1}\Theta(\mathrm{R}_i\mathrm{R}_{i+1}, \mathrm{R}_{i+j}\mathrm{R}_{i+j+1}) \quad (j = 1, 2, \cdots, \lambda; \lambda < L) \tag{16}$$

which represents the j-tier structural correlation factor between all the j-th most contiguous dinucleotides. The correlation function $\Theta(\mathrm{R}_i\mathrm{R}_{i+1}, \mathrm{R}_{i+j}\mathrm{R}_{i+j+1})$ is defined by

$$\Theta(\mathrm{R}_i\mathrm{R}_{i+1}, \mathrm{R}_{i+j}\mathrm{R}_{i+j+1}) = \frac{1}{\mu}\sum_{v=1}^{\mu}[P_v(\mathrm{R}_i\mathrm{R}_{i+1}) - P_v(\mathrm{R}_{i+j}\mathrm{R}_{i+j+1})]^2 \tag{17}$$

where μ is the number of physicochemical indices, in this study, 6 indices reflecting the local DNA structural properties (8) (**Table 3**) are employed to generate the PseKNC feature vector; $P_v(\mathrm{R}_i\mathrm{R}_{i+1})$ ($P_v(\mathrm{R}_{i+j}\mathrm{R}_{i+j+1})$) represents the numerical value of the v-th ($v = 1, 2, \cdots, \mu$) physicochemical index for the dinucleotide $\mathrm{R}_i\mathrm{R}_{i+1}$ ($\mathrm{R}_{i+j}\mathrm{R}_{i+j+1}$) at position i (i+j).

For more information about this approach, please refer to (9,10).

1.3.3 General parallel correlation pseudo dinucleotide composition (PC-PseDNC-General)

In PC-PseDNC-General (11) approach, the users cannot only select the 148 built-in physiochemical indices (**Table 1**), but also can upload their own indices to generate the PC-PseDNC-General feature vector.

Given a DNA sequence **D** (**Eq. 1**), the PC-PseDNC-General feature vector of **D** is defined:

$$\mathbf{D} = \begin{bmatrix} d_1 & d_2 & \cdots & d_{16} & d_{16+1} & \cdots & d_{16+\lambda} \end{bmatrix}^{\mathrm{T}} \tag{18}$$

where

$$d_k = \begin{cases} \dfrac{f_k}{\sum_{i=1}^{16} f_i + w\sum_{j=1}^{\lambda}\theta_j} & (1 \le k \le 16) \\ \dfrac{w\theta_{k-16}}{\sum_{i=1}^{16} f_i + w\sum_{j=1}^{\lambda}\theta_j} & (16 + 1 \le k \le 16 + \lambda) \end{cases} \tag{19}$$

where f_k（k=1,2,⋯,16) is the normalized occurrence frequency of dinucleotides in the DNA sequence; the parameter λ is an integer, representing the highest counted rank (or tier) of the correlation along a DNA sequence; w is the weight factor ranging from

0 to 1; θ_j (j=1, 2, ⋯, λ) is called the j-tier correlation factor that reflects the sequence-order correlation between all the most contiguous dinucleotides along a DNA sequence, which is defined:

$$\begin{cases} \theta_1 = \frac{1}{L\text{-}2}\sum_{i=1}^{L-2}\Theta(\mathrm{R}_i\mathrm{R}_{i+1}, \mathrm{R}_{i+1}\mathrm{R}_{i+2}) \\ \theta_2 = \frac{1}{L\text{-}3}\sum_{i=1}^{L-3}\Theta(\mathrm{R}_i\mathrm{R}_{i+1}, \mathrm{R}_{i+2}\mathrm{R}_{i+3}) \\ \theta_3 = \frac{1}{L\text{-}4}\sum_{i=1}^{L-4}\Theta(\mathrm{R}_i\mathrm{R}_{i+1}, \mathrm{R}_{i+3}\mathrm{R}_{i+4}) \qquad (\lambda < L-1) \\ \ldots\ldots \\ \theta_\lambda = \frac{1}{L\text{-}1\text{-}\lambda}\sum_{i=1}^{L-1-\lambda}\Theta(\mathrm{R}_i\mathrm{R}_{i+1}, \mathrm{R}_{i+\lambda}\mathrm{R}_{i+\lambda+1}) \end{cases} \tag{20}$$

where the correlation function is given by

$$\Theta(\mathrm{R}_i\mathrm{R}_{i+1}, \mathrm{R}_j\mathrm{R}_{j+1}) = \frac{1}{\mu}\sum_{u=1}^{\mu}[P_u(\mathrm{R}_i\mathrm{R}_{i+1}) - P_u(\mathrm{R}_j\mathrm{R}_{j+1})]^2 \tag{21}$$

where μ is the number of physicochemical indices listed in the **Table 1**; $P_u(\mathrm{R}_i\mathrm{R}_{i+1})$ ($P_u(\mathrm{R}_j\mathrm{R}_{j+1})$) represents the numerical value of the u-th ($u = 1,2,\cdots,\mu$) physicochemical index for the dinucleotide $\mathrm{R}_i\mathrm{R}_{i+1}$ ($\mathrm{R}_j\mathrm{R}_{j+1}$) at position i (j).

1.3.4 General parallel correlation pseudo trinucleotide composition (PC-PseTNC-General)

In PC-PseTNC-General (11) approach, the users cannot only select the 12 built-in physiochemical indices (**Table 2**), but also can upload their own indices to generate the PC-PseTNC-General feature vector.

Given a DNA sequence **D** (**Eq. 1**), the PC-PseTNC-General feature vector of **D** is defined:

$$\mathbf{D} = \begin{bmatrix} d_1 & d_2 & \cdots & d_{64} & d_{64+1} & \cdots & d_{64+\lambda} \end{bmatrix}^{\mathbf{T}} \tag{22}$$

where

$$d_k = \begin{cases} \frac{f_k}{\sum_{i=1}^{64} f_i + w\sum_{j=1}^{\lambda}\theta_j} & (1 \le k \le 64) \\ \frac{w\theta_{k-64}}{\sum_{i=1}^{64} f_i + w\sum_{j=1}^{\lambda}\theta_j} & (64+1 \le k \le 64+\lambda) \end{cases} \tag{23}$$

where f_k（k=1,2,⋯,64) is the normalized occurrence frequency of trinucleotide in the DNA sequence; the parameter λ is an integer, representing the highest counted rank (or tier) of the correlation along a DNA sequence; w is the weight factor ranging from 0 to 1; θ_j (j=1, 2, ⋯, λ) is called the j-tier correlation factor that reflects the sequence-order correlation between all the most contiguous trinucleotides along a DNA sequence, which is defined:

$$\begin{cases} \theta_1 = \dfrac{1}{L\text{-}3}\sum_{i=1}^{L-3}\Theta(\mathrm{R}_i\mathrm{R}_{i+1}\,\mathrm{R}_{i+2},\mathrm{R}_{i+1}\mathrm{R}_{i+2}\mathrm{R}_{i+3}) \\ \theta_2 = \dfrac{1}{L\text{-}4}\sum_{i=1}^{L-4}\Theta(\mathrm{R}_i\mathrm{R}_{i+1}\,\mathrm{R}_{i+2},\mathrm{R}_{i+2}\mathrm{R}_{i+3}\mathrm{R}_{i+4}) \\ \theta_3 = \dfrac{1}{L\text{-}5}\sum_{i=1}^{L-5}\Theta(\mathrm{R}_i\mathrm{R}_{i+1}\,\mathrm{R}_{i+2},\mathrm{R}_{i+3}\mathrm{R}_{i+4}\mathrm{R}_{i+5}) \qquad (\lambda < L-2) \\ \dots\dots \\ \theta_\lambda = \dfrac{1}{L\text{-}2\text{-}\lambda}\sum_{i=1}^{L-2-\lambda}\Theta(\mathrm{R}_i\mathrm{R}_{i+1}\,\mathrm{R}_{i+2},\mathrm{R}_{i+\lambda}\mathrm{R}_{i+\lambda+1}\mathrm{R}_{i+\lambda+2}) \end{cases} \tag{24}$$

where the correlation function is given by

$$\Theta(\mathrm{R}_i\mathrm{R}_{i+1}\mathrm{R}_{i+2},\mathrm{R}_j\mathrm{R}_{j+1}\mathrm{R}_{j+2}) = \frac{1}{\mu}\sum_{u=1}^{\mu}[P_u(\mathrm{R}_i\mathrm{R}_{i+1}\mathrm{R}_{i+2}) - P_u(\mathrm{R}_j\mathrm{R}_{j+1}\mathrm{R}_{j+2})]^2 \tag{25}$$

where μ is the number of physicochemical indices considered that are listed in the **Table 2**; $P_u(\mathrm{R}_i\mathrm{R}_{i+1}\mathrm{R}_{i+2})$ $(P_u(\mathrm{R}_j\mathrm{R}_{j+1}\,\mathrm{R}_{j+2}))$ represents the numerical value of the *u*-th $(u = 1, 2, \cdots, \mu)$ physicochemical index for the tri-nucleotide $\mathrm{R}_i\mathrm{R}_{i+1}\mathrm{R}_{i+2}$ $(\mathrm{R}_j\mathrm{R}_{j+1}\mathrm{R}_{j+2})$ at position *i* (*j*).

1.3.5 General series correlation pseudo dinucleotide composition (SC-PseDNC-General)

SC-PseDNC-General (11) is a variant of PC-PseDNC-General, which differs in the equations of calculating the correlation factors reflecting the sequence-order correlation between all the most contiguous dinucleotides along a DNA sequence.

Given a DNA sequence **D** (**Eq. 1**), the SC-PseDNC-General feature vector of **D** is defined:

$$\mathbf{D} = [d_1 \quad d_2 \quad \cdots \quad d_{16} \quad d_{16+1} \quad \cdots \quad d_{16+\lambda} \quad d_{16+\lambda+1} \quad \cdots \quad d_{16+\lambda\Lambda}]^{\mathbf{T}} \tag{26}$$

where

$$d_k = \begin{cases} \dfrac{f_k}{\sum_{i=1}^{16} f_i + w\sum_{j=1}^{\lambda\Lambda}\theta_j} & (1 \le k \le 16) \\ \dfrac{w\theta_{k-16}}{\sum_{i=1}^{16} f_i + w\sum_{j=1}^{\lambda\Lambda}\theta_j} & (17 \le k \le 16+\lambda\Lambda) \end{cases} \tag{27}$$

where f_k（k=1, 2, ⋯, 16) is the normalized occurrence frequency of dinucleotide in the DNA sequence; the parameter λ is an integer, representing the highest counted rank (or tier) of the correlation along a DNA sequence; *w* is the weight factor ranging from 0 to 1; Λ is the number of physicochemical indices (**Table 1**); θ_j (j = 1, 2, ⋯, λ) is called the *j*-tier correlation factor that reflects the sequence-order correlation between all the most contiguous dinucleotides along a DNA sequence, which is defined:

$$\begin{cases} \theta_1 = \frac{1}{L\text{-}3}\sum_{i=1}^{L-3} \mathrm{J}_{i,i+1}^{1} \\ \theta_2 = \frac{1}{L\text{-}3}\sum_{i=1}^{L-3} \mathrm{J}_{i,i+1}^{2} \\ \cdots\cdots \\ \theta_\Lambda = \frac{1}{L\text{-}3}\sum_{i=1}^{L-3} \mathrm{J}_{i,i+1}^{\Lambda} \qquad \lambda < (L-2) \\ \cdots\cdots \\ \theta_{\lambda\Lambda-1} = \frac{1}{L\text{-}\lambda\text{-}2}\sum_{i=1}^{L-\lambda-2} \mathrm{J}_{i,i+\lambda}^{\Lambda-1} \\ \theta_{\lambda\Lambda} = \frac{1}{L\text{-}\lambda\text{-}2}\sum_{i=1}^{L-\lambda-2} \mathrm{J}_{i,i+\lambda}^{\Lambda} \end{cases} \tag{28}$$

The correlation function is given by

$$\mathrm{J}_{i,i+m}^{u} = P_u(\mathrm{R}_i\mathrm{R}_{i+1}) \cdot P_u(\mathrm{R}_{i+m}\mathrm{R}_{i+\mathrm{m}+1}) \quad (u = 1, 2, \cdots, \Lambda;\ m=1,2,\cdots,\lambda;\ i=1,2,\cdots,L\text{-}m\text{-}1) \tag{29}$$

where $P_u(\mathrm{R}_i\mathrm{R}_{i+1})$ ($P_u(\mathrm{R}_{i+m}\mathrm{R}_{i+m+1})$) represents the numerical value of the u-th ($u = 1,\ 2,\ \cdots,\ \mu$) physiochemical index for the dinucleotide $\mathrm{R}_i\mathrm{R}_{i+1}$ ($\mathrm{R}_{i+m}\mathrm{R}_{i+\mathrm{m}+1}$) at position i ($i+m$).

1.3.6 General series correlation pseudo trinucleotide composition (SC-PseTNC-General)

SC-PseTNC-General (11) is a variant of PC-PseTNC-General, which differs in the equations of calculating the correlation factors reflecting the sequence-order correlation between all the most contiguous dinucleotides along a DNA sequence.

Given a DNA sequence **D** (**Eq. 1**), the SC-PseTNC-General feature vector of **D** is defined:

$$\mathbf{D} = \begin{bmatrix} d_1 & d_2 & \cdots & d_{64} & d_{64+1} & \cdots & d_{64+\lambda} & d_{64+\lambda+1} & \cdots & d_{64+\lambda\Lambda} \end{bmatrix}^{\mathbf{T}} \tag{30}$$

where

$$d_k = \begin{cases} \dfrac{f_k}{\sum_{i=1}^{64} f_i + w\sum_{j=1}^{\lambda\Lambda}\theta_j} & (1 \le k \le 64) \\ \dfrac{w\theta_{k-64}}{\sum_{i=1}^{64} f_i + w\sum_{j=1}^{\lambda\Lambda}\theta_j} & (64+1 \le k \le 64+\lambda\Lambda) \end{cases} \tag{31}$$

where f_k（k=1, 2, ···, 64) is the normalized occurrence frequency of trinucleotide in the DNA sequence; the parameter λ is an integer, representing the highest counted rank (or tier) of the correlation along a DNA sequence; w is the weight factor ranging from 0 to 1; Λ is the number of physicochemical indices (**Table 2**); θ_j (j = 1, 2, ···, λ) is called the j-tier correlation factor reflecting the sequence-order correlation between all the most contiguous trinucleotides along a DNA sequence, which is defined:

$$
\begin{cases}
\theta_1 = \dfrac{1}{L\text{-}4}\sum_{i=1}^{L-4} \mathrm{J}_{i,i+1}^{1} \\
\theta_2 = \dfrac{1}{L\text{-}4}\sum_{i=1}^{L-4} \mathrm{J}_{i,i+1}^{2} \\
\cdots\cdots \\
\theta_\Lambda = \dfrac{1}{L\text{-}4}\sum_{i=1}^{L-4} \mathrm{J}_{i,i+1}^{\Lambda} \qquad \lambda < (L-3) \\
\cdots\cdots \\
\theta_{\lambda\Lambda-1} = \dfrac{1}{L\text{-}\lambda\text{-}3}\sum_{i=1}^{L-\lambda-3} \mathrm{J}_{i,i+\lambda}^{\Lambda-1} \\
\theta_{\lambda\Lambda} = \dfrac{1}{L\text{-}\lambda\text{-}3}\sum_{i=1}^{L-\lambda-3} \mathrm{J}_{i,i+\lambda}^{\Lambda}
\end{cases}
\tag{32}
$$

The correlation function is given by

$$
\begin{cases}
\mathrm{J}_{i,i+m}^{u} = P_u(\mathrm{R}_i\mathrm{R}_{i+1}\mathrm{R}_{i+2})\cdot P_u(\mathrm{R}_{i+m}\mathrm{R}_{i+m+1}\mathrm{R}_{i+m+2}) \\
u = 1,2,\cdots,\Lambda;\ m=1,2,\cdots,\lambda;\ i=1,2,\cdots,L\text{-}m\text{-}2
\end{cases}
\tag{33}
$$

where $P_u(\mathrm{R}_i\mathrm{R}_{i+1}\mathrm{R}_{i+2})$ ($P_u(\mathrm{R}_{i+m}\mathrm{R}_{i+m+1}\mathrm{R}_{i+m+2})$) represents the numerical value of the *u*-th ($u = 1,\ 2,\ \cdots,\ \mu$) physiochemical index for the tri-nucleotide $\mathrm{R}_i\mathrm{R}_{i+1}\mathrm{R}_{i+2}$ ($\mathrm{R}_{i+m}\mathrm{R}_{i+m+1}\mathrm{R}_{i+m+2}$) at position *i* (*i*+*m*).

2. RNA

2.1 Ribonucleic acid composition

2.1.1 Basic kmer (Kmer)

Basic kmer (12) is the simplest approach to represent the RNAs, in which the RNA sequences are represented as the occurrence frequencies of *k* neighboring nucleic acids.

2.2 Autocorrelation

2.2.1 Dinucleotide-based auto covariance (DAC)

Suppose an RNA sequence **R** with *L* nucleic acid residues; i.e.

$$
\mathrm{R} = \mathrm{R}_1\mathrm{R}_2\mathrm{R}_3\mathrm{R}_4\mathrm{R}_5\mathrm{R}_6\mathrm{R}_7\cdots\mathrm{R}_L \tag{34}
$$

where R_1 represents the nucleic acid residue at the sequence position 1, R_2 the nucleic acid residue at position 2, and so forth.

The DAC (4-6) measures the correlation of the same physicochemical index between two dinucleotides separated by a distance of *lag* along the sequence, which can be calculated as:

$$\mathrm{DAC}(u, lag) = \sum_{i=1}^{L-lag-1} (P_u(\mathrm{R}_i\mathrm{R}_{i+1}) - \overline{P}_u)(P_u(\mathrm{R}_{i+lag}\mathrm{R}_{i+lag+1}) - \overline{P}_u) / (L - lag - 1) \quad (35)$$

where u is a physicochemical index; L is the length of the RNA sequence, $P_u(\mathrm{R}_i\mathrm{R}_{i+1})$ ($P_u(\mathrm{R}_{i+lag}\mathrm{R}_{i+lag+1})$) means the numerical value of the physicochemical index u for the dinucleotide $\mathrm{R}_i\mathrm{R}_{i+1}$ ($\mathrm{R}_{i+lag}\mathrm{R}_{i+lag+1}$) at position i ($i+lag$), $\overline{P}_u$ is the average value for physicochemical index u along the whole sequence:

$$\overline{P_u} = \sum_{j=1}^{L-1} P_u(\mathrm{R}_j\mathrm{R}_{j+1}) / (L-1) \quad (36)$$

In such a way, the length of DAC feature vector is $N*$LAG, where N is the number of physicochemical indices (**Table 4**), which are extracted from (6,7), and LAG is the maximum of *lag* (*lag* = 1, 2, … , LAG).

2.2.2 Dinucleotide-based cross covariance (DCC)

Given an RNA sequence **R** (**Eq. 34**), the DCC (4,6) approach measures the correlation of two different physicochemical indices between two dinucleotides separated by *lag* nucleic acids along the sequence, which can be calculated by:

$$\mathrm{DCC}(u_1, u_2, lag) = \sum_{i=1}^{L-lag-1} (P_{u_1}(R_iR_{i+1}) - \overline{P}_{u_1})(P_{u_2}(R_{i+lag}R_{i+lag+1}) - \overline{P}_{u_2}) / (L - lag - 1) \quad (37)$$

where u_1, u_2 are two different physicochemical indices, L is the length of the RNA sequence, $P_{u_1}(\mathrm{R}_i\mathrm{R}_{i+1})$ ($P_{u_2}(\mathrm{R}_i\mathrm{R}_{i+1})$) is the numerical value of the physicochemical index u_1 (u_2) for the dinucleotide $\mathrm{R}_i\mathrm{R}_{i+1}$ at position i, $\overline{P}_{u_1}$ ($\overline{P}_{u_2}$) is the average value for physicochemical index value u_1 (u_2) along the whole sequence:

$$\overline{P_u} = \sum_{j=1}^{L-1} P_u(\mathrm{R}_j\mathrm{R}_{j+1}) / (L-1) \quad (38)$$

In such a way, the length of the DCC feature vector is N*(N-1)*LAG, where N is the number of physicochemical indices (**Table 4**) and LAG is the maximum of *lag* (*lag*=1, 2, …, LAG).

2.2.3 Dinucleotide-based auto-cross covariance (DACC)

DACC (4,6) is a combination of DAC and DCC. Therefore, the length of the DACC feature vector is N*N*LAG, where N is the number of physicochemical indices (**Table 4**) and LAG is the maximum of *lag* (*lag* = 1, 2, …, LAG).

2.3 Pseudo ribonucleic acid composition

2.3.1 General parallel correlation pseudo dinucleotide composition (PC-PseDNC-General)

In PC-PseDNC-General (6) approach, the users cannot only select the 22 built-in physiochemical indices (**Table 4**), but also can upload their own indices to generate the PC-PseDNC-General feature vector.

Given an RNA sequence **R** (**Eq. 34**), the PC-PseDNC-General feature vector of **R** is defined:

$$\mathbf{R} = \begin{bmatrix} d_1 & d_2 & \cdots & d_{16} & d_{16+1} & \cdots & d_{16+\lambda} \end{bmatrix}^{\mathrm{T}} \quad (39)$$

where

$$d_k = \begin{cases} \dfrac{f_k}{\sum_{i=1}^{16} f_i + w\sum_{j=1}^{\lambda}\theta_j} & (1 \le k \le 16) \\ \dfrac{w\theta_{k-16}}{\sum_{i=1}^{16} f_i + w\sum_{j=1}^{\lambda}\theta_j} & (16+1 \le k \le 16+\lambda) \end{cases} \quad (40)$$

where f_k (k=1,2,⋯,16) is the normalized occurrence frequency of dinucleotide in the RNA sequence; the parameter λ is an integer, representing the highest counted rank (or tier) of the correlation along a RNA sequence; w is the weight factor ranging from 0 to 1; θ_j (j=1, 2, ⋯, λ) is called the j-tier correlation factor reflecting the sequence-order correlation between all the i-th most contiguous dinucleotides along an RNA sequence, which is defined:

$$\begin{cases} \theta_1 = \dfrac{1}{L\text{-}2}\sum_{i=1}^{L-2}\Theta(\mathrm{R}_i\mathrm{R}_{i+1}, \mathrm{R}_{i+1}\mathrm{R}_{i+2}) \\ \theta_2 = \dfrac{1}{L\text{-}3}\sum_{i=1}^{L-3}\Theta(\mathrm{R}_i\mathrm{R}_{i+1}, \mathrm{R}_{i+2}\mathrm{R}_{i+3}) \\ \theta_3 = \dfrac{1}{L\text{-}4}\sum_{i=1}^{L-4}\Theta(\mathrm{R}_i\mathrm{R}_{i+1}, \mathrm{R}_{i+3}\mathrm{R}_{i+4}) \\ \cdots\cdots \\ \theta_\lambda = \dfrac{1}{L\text{-}1\text{-}\lambda}\sum_{i=1}^{L-1-\lambda}\Theta(\mathrm{R}_i\mathrm{R}_{i+1}, \mathrm{R}_{i+\lambda}\mathrm{R}_{i+\lambda+1}) \end{cases} \quad (\lambda < L) \quad (41)$$

where the correlation function is given by

$$\Theta(\mathrm{R}_i\mathrm{R}_{i+1}, \mathrm{R}_j\mathrm{R}_{j+1}) = \frac{1}{\mu}\sum_{u=1}^{\mu}[P_u(\mathrm{R}_i\mathrm{R}_{i+1}) - P_u(\mathrm{R}_j\mathrm{R}_{j+1})]^2 \quad (42)$$

where μ is the number of physicochemical indices considered that are listed in the **Table 4**; $P_u(\mathrm{R}_i\mathrm{R}_{i+1})$ ($P_u(\mathrm{R}_j\mathrm{R}_{j+1})$) represents the numerical value of the u-th ($u = 1, 2, \cdots, \mu$) physicochemical index for the dinucleotide $\mathrm{R}_i\mathrm{R}_{i+1}$($\mathrm{R}_j\mathrm{R}_{j+1}$) at position i (j).

2.3.2 General series correlation pseudo dinucleotide composition (SC-PseDNC-General)

SC-PseDNC-General (6) is a variant of PC-PseDNC-General, which differs in the equations of calculating the correlation factors reflecting the sequence-order correlation between all the most contiguous dinucleotides along an RNA sequence.

Given an RNA sequence **R** (**Eq. 34**), the SC-PseDNC-General feature vector of **R** is defined:

$$\mathbf{R} = \begin{bmatrix} d_1 & d_2 & \cdots & d_{16} & d_{16+1} & \cdots & d_{16+\lambda} & d_{16+\lambda+1} & \cdots & d_{16+\lambda\Lambda} \end{bmatrix}^{\mathbf{T}} \quad (43)$$

where

$$d_k = \begin{cases} \dfrac{f_k}{\sum_{i=1}^{16} f_i + w\sum_{j=1}^{\lambda\Lambda}\theta_j} & (1 \le k \le 16) \\ \dfrac{w\theta_{k-16}}{\sum_{i=1}^{16} f_i + w\sum_{j=1}^{\lambda\Lambda}\theta_j} & (16+1 \le k \le 16+\lambda\Lambda) \end{cases} \quad (44)$$

where f_k (k=1, 2, ⋯, 16) is the normalized occurrence frequency of dinucleotides in the RNA sequence; the parameter λ is an integer, representing the highest counted rank (or tier) of the correlation along an RNA sequence; w is the weight factor ranging from 0 to 1; Λ is the number of physicochemical indices (**Table 4**); θ_j (j = 1, 2, ⋯, λ) is called the j-tier correlation factor reflecting the sequence-order correlation between all the j-th most contiguous dinucleotides along an RNA sequence, which is defined:

$$\begin{cases} \theta_1 = \dfrac{1}{L\text{-}3}\sum_{i=1}^{L-3} \mathrm{J}_{i,i+1}^{1} \\ \theta_2 = \dfrac{1}{L\text{-}3}\sum_{i=1}^{L-3} \mathrm{J}_{i,i+1}^{2} \\ \cdots\cdots \\ \theta_\Lambda = \dfrac{1}{L\text{-}3}\sum_{i=1}^{L-3} \mathrm{J}_{i,i+1}^{\Lambda} & \lambda < (L-2) \\ \cdots\cdots \\ \theta_{\lambda\Lambda-1} = \dfrac{1}{L\text{-}\lambda\text{-}2}\sum_{i=1}^{L-\lambda-2} \mathrm{J}_{i,i+\lambda}^{\Lambda-1} \\ \theta_{\lambda\Lambda} = \dfrac{1}{L\text{-}\lambda\text{-}2}\sum_{i=1}^{L-\lambda-2} \mathrm{J}_{i,i+\lambda}^{\Lambda} \end{cases} \quad (45)$$

The correlation function is given by

$$\begin{cases} \mathrm{J}_{i,i+m}^{u} = P_u(\mathrm{R}_i\mathrm{R}_{i+1}) \cdot P_u(\mathrm{R}_{i+m}\mathrm{R}_{i+m+1}) \\ u = 1,2,\cdots,\Lambda;\ m=1,2,\cdots,\lambda;\ i=1,2,\cdots,L\text{-}\lambda\text{-}2 \end{cases} \quad (46)$$

$P_u(\mathrm{R}_i\mathrm{R}_{i+1})$ ($P_u(\mathrm{R}_{i+m}\mathrm{R}_{i+\mathrm{m}+1})$) represents the numerical value of the u-th ($u = 1,\ 2,\ \cdots,\ \mu$) physiochemical index for the dinucleotide $\mathrm{R}_i\mathrm{R}_{i+1}$ ($\mathrm{R}_{i+m}\mathrm{R}_{i+\mathrm{m}+1}$) at position i (i+m).

3. Protein

3.1 Amino acid composition

3.1.1 Basic kmer (Kmer)

Basic kmer (13) is the simplest approach to represent the proteins, in which the protein sequences are represented as the occurrence frequencies of *k* neighboring amino acids.

3.2 Autocorrelation

3.2.1 Auto covariance (AC)

Suppose a protein sequence **P** with *L* amino acid residues; i.e.

$$\mathrm{P} = \mathrm{R}_1\mathrm{R}_2\mathrm{R}_3\mathrm{R}_4\mathrm{R}_5\mathrm{R}_6\mathrm{R}_7 \cdots \mathrm{R}_L \tag{47}$$

where R_1 represents the amino acid residue at the sequence position 1, R_2 the amino acid residue at position 2 and so forth.

The AC (4,5,14) approach measures the correlation of the same property between two residues separated by a distance of *lag* along the sequence, which can be calculated as:

$$\mathrm{AC}(i, lag) = \sum_{i=1}^{L-lag} (P_u(\mathrm{R}_i) - \overline{P}_u)(P_u(\mathrm{R}_{i+lag}) - \overline{P}_u) / (L - lag) \tag{48}$$

where *u* is a physicochemical index, *L* is the length of the protein sequence, $P_u(\mathrm{R}_i)$ means the numerical value of the physicochemical index *u* for the amino acid R_i at position *i*, $\overline{P}_u$ is the average value for physicochemical index *u* along the whole sequence:

$$\overline{P_u} = \sum_{j=1}^{L} P_u(\mathrm{R}_j) / L \tag{49}$$

In such a way, the length of AC feature vector is $N*$LAG, where *N* is the number of physicochemical indices (**Table 5**) extracted from AAindex (15); LG is the maximum of *lag* (*lag*=1,2,...,LG).
For more information of this approach, please refer to (4,5).

3.2.2 Cross covariance (CC)

Given a protein sequence **P** (**Eq.47**), the CC (4,5,14) approach measures the correlation of two different properties between two residues separated by a distance of *lag* along the sequence, which can be calculated by:

$$\mathrm{CC}(u_1, u_2, lag) = \sum_{i=1}^{L-lag} (P_{u_1}(R_i) - \overline{P}_{u_1})(P_{u_2}(R_{i+lag}) - \overline{P}_{u_2}) / (L - lag) \tag{50}$$

where u_1, u_2 are two different physicochemical indices, *L* is the length of the protein sequence, $P_{u_1}(\mathrm{R}_i)$ ($P_{u_2}(\mathrm{R}_{i+lag})$) is the numerical value of the physicochemical index u_1 (u_2) for the amino acid R_i (R_{i+lag}) at position *i* (*i*+*lag*), $\overline{P}_{u_1}$ ($\overline{P}_{u_2}$) is the average value for physicochemical index value u_1 (u_2) along the whole sequence:

$$\overline{P_u} = \sum_{j=1}^{L} P_u(\mathrm{R}_j)/L \tag{51}$$

In such a way, the length of the CC feature vector is *N**(*N*-1)*LAG, where *N* is the number of physicochemical indices (**Table 5**) and LAG is the maximum of *lag* (*lag*=1, 2, …, LAG).

For more information of this approach, please refer to (4,5).

3.2.3 Auto-cross covariance (ACC)

ACC (4,5,14) is a combination of AC and CC. Therefore, the length of the ACC feature vector is *N**N*LAG, where *N* is the number of physicochemical indices (**Table 5**) and LAG is the maximum of *lag* (*lag* = 1, 2, …, LAG).

3.3 Pseudo amino acid composition

3.3.1 Parallel correlation pseudo amino acid composition (PC-PseAAC)

PC-PseAAC (16) is an approach incorporating the contiguous local sequence-order information and the global sequence-order information into the feature vector of the protein sequence.

Given a Protein sequence **P** (**Eq.47**), the PC-PseAAC feature vector of **P** is defined:

$$\mathbf{P} = \begin{bmatrix} x_1 & x_2 & \cdots & x_{20} & x_{20+1} & \cdots & x_{20+\lambda} \end{bmatrix}^{\mathbf{T}} \tag{52}$$

where

$$x_u = \begin{cases} \dfrac{f_u}{\sum_{i=1}^{20} f_i + w\sum_{j=1}^{\lambda}\theta_j} & (1 \le u \le 20) \\ \dfrac{w\theta_{u-20}}{\sum_{i=1}^{20} f_i + w\sum_{j=1}^{\lambda}\theta_j} & (20+1 \le u \le 20+\lambda) \end{cases} \tag{53}$$

where f_i (i=1,2,···,20) is the normalized occurrence frequency of the 20 amino acids in the protein **P**; the parameter λ is an integer, representing the highest counted rank (or tier) of the correlation along a protein sequence; *w* is the weight factor ranging from 0 to 1; θ_j (j=1,2,···,λ) is called the *j*-tier correlation factor reflecting the sequence-order correlation between all the *j*-th most contiguous residues along a protein chain, which is defined:

$$\begin{cases} \theta_1 = \dfrac{1}{L\text{-}1}\sum_{i=1}^{L-1}\Theta(\mathrm{R}_i, \mathrm{R}_{i+1}) \\ \theta_2 = \dfrac{1}{L\text{-}2}\sum_{i=1}^{L-2}\Theta(\mathrm{R}_i, \mathrm{R}_{i+2}) \\ \theta_3 = \dfrac{1}{L\text{-}3}\sum_{i=1}^{L-3}\Theta(\mathrm{R}_i, \mathrm{R}_{i+3}) \qquad (\lambda < L) \\ \cdots\cdots \\ \theta_\lambda = \dfrac{1}{L\text{-}\lambda}\sum_{i=1}^{L-\lambda}\Theta(\mathrm{R}_i, \mathrm{R}_{i+\lambda}) \end{cases} \tag{54}$$

where the correlation function is given by

$$\Theta(\mathrm{R}_i, \mathrm{R}_j) = \frac{1}{3}\left\{\left[H_1(\mathrm{R}_j) - H_1(\mathrm{R}_i)\right]^2 + \left[H_2(\mathrm{R}_j) - H_2(\mathrm{R}_i)\right]^2 + \left[M(\mathrm{R}_j) - M(\mathrm{R}_i)\right]^2\right\} \tag{55}$$

where $H_1(\mathrm{R}_i)$, $H_2(\mathrm{R}_i)$, and $M(\mathrm{R}_i)$ are, respectively, the hydrophobicity value, hydrophilicity value, and side-chain mass (**Table 6**) of the amino acid R_i; Note that before substituting the values of hydrophobicity, hydrophilicity, and side-chain mass into **Eq. 55**, they are all subjected to a standard conversion as described by the following equation:

$$\begin{cases} H_1(i) = \dfrac{H_1^0(i) - \sum_{i=1}^{20}\dfrac{H_1^0(i)}{20}}{\sqrt{\dfrac{\sum_{i=1}^{20}\left[H_1^0(i) - \sum_{i=1}^{20}\dfrac{H_1^0(i)}{20}\right]^2}{20}}} \\ H_2(i) = \dfrac{H_2^0(i) - \sum_{i=1}^{20}\dfrac{H_2^0(i)}{20}}{\sqrt{\dfrac{\sum_{i=1}^{20}\left[H_2^0(i) - \sum_{i=1}^{20}\dfrac{H_2^0(i)}{20}\right]^2}{20}}} \\ M(i) = \dfrac{M^0(i) - \sum_{i=1}^{20}\dfrac{M^0(i)}{20}}{\sqrt{\dfrac{\sum_{i=1}^{20}\left[M^0(i) - \sum_{i=1}^{20}\dfrac{M^0(i)}{20}\right]^2}{20}}} \end{cases} \tag{56}$$

where $H_1^0(i)$ is the original hydrophobicity value of the i-th amino acid; $H_2^0(i)$ the corresponding original hydrophilicity value; $M^0(i)$ the mass of the i-th amino acid side chain.

3.3.2 Series correlation pseudo amino acid composition (SC-PseAAC)

SC-PseAAC (17) is a variant of PC-PseAAC. Given a protein sequence **P** (**Eq.47**), the SC-PseAAC feature vector of **P** is defined:

$$\mathbf{P} = \left[p_1 \quad p_2 \quad \cdots \quad p_{20} \quad p_{20+1} \quad \cdots \quad p_{20+\lambda} \quad p_{20+\lambda+1} \quad \cdots \quad p_{20+2\lambda}\right]^{\mathbf{T}} \tag{57}$$

where

$$p_u = \begin{cases} \dfrac{f_u}{\sum_{i=1}^{20} f_i + w\sum_{j=1}^{2\lambda} \tau_j} & (1 \le u \le 20) \\ \dfrac{w\tau_{u-20}}{\sum_{i=1}^{20} f_i + w\sum_{j=1}^{2\lambda} \tau_j} & (20+1 \le u \le 20+2\lambda) \end{cases} \quad (58)$$

where f_i *(i* = 1, 2,... , 20*)* is the normalized occurrence frequency of the 20 native amino acids in the protein **P**; the parameter λ is an integer, representing the highest counted rank (or tier) of the correlation along a protein sequence; *w* is the weight factor ranging from 0 to 1; τ_j the *j* -tier sequence-correlation factor that reflects the sequence-order correlation between all the most contiguous residues along a protein sequence, which is defined:

$$\begin{cases} \tau_1 = \dfrac{1}{L\text{-}1}\sum_{i=1}^{L-1} H_{i,i+1}^1 \\ \tau_2 = \dfrac{1}{L\text{-}1}\sum_{i=1}^{L-1} H_{i,i+1}^2 \\ \tau_3 = \dfrac{1}{L\text{-}2}\sum_{i=1}^{L-2} H_{i,i+2}^1 \\ \tau_4 = \dfrac{1}{L\text{-}2}\sum_{i=1}^{L-2} H_{i,i+2}^2 \\ \ldots\ldots \\ \tau_{2\lambda-1} = \dfrac{1}{L\text{-}\lambda}\sum_{i=1}^{L-\lambda} H_{i,i+\lambda}^1 \\ \tau_{2\lambda} = \dfrac{1}{L\text{-}\lambda}\sum_{i=1}^{L-\lambda} H_{i,i+\lambda}^2 \end{cases} \quad \lambda < L-1 \quad (59)$$

where $H_{i,\mathrm{j}}^1$ and $H_{i,\mathrm{j}}^2$ are the hydrophobicity and hydrophilicity correlation functions given by

$$\begin{cases} H_{i,j}^1 = h^1(\mathrm{R}_i)\cdot h^1(\mathrm{R}_j) \\ H_{i,j}^2 = h^2(\mathrm{R}_i)\cdot h^2(\mathrm{R}_j) \end{cases} \quad (60)$$

where $h^1(\mathrm{R}_i)$ and $h^2(\mathrm{R}_i)$ are, respectively, the hydrophobicity and hydrophilicity values(**Table 7**) for the *i*-th (*i* = 1, 2, . . . ,*L*) amino acid in **Eq.47**, and the dot (•) means the multiplication sign.

Note that before substituting the values of hydrophobicity and hydrophilicity into **Eq.60**, they are all subjected to a standard conversion as described by the following equation:

$$\begin{cases} h^1(\mathrm{R}_i) = \dfrac{h_0^1(\mathrm{R}_i) - \sum_{k=1}^{20} \dfrac{h_0^1(\mathbb{R}_k)}{20}}{\sqrt{\dfrac{\sum_{u=1}^{20}\left[h_0^1(\mathbb{R}_u) - \sum_{k=1}^{20} \dfrac{h_0^1(\mathbb{R}_k)}{20}\right]^2}{20}}} \\ h^2(\mathrm{R}_i) = \dfrac{h_0^2(\mathrm{R}_i) - \sum_{k=1}^{20} \dfrac{h_0^2(\mathbb{R}_k)}{20}}{\sqrt{\dfrac{\sum_{u=1}^{20}\left[h_0^2(\mathbb{R}_u) - \sum_{k=1}^{20} \dfrac{h_0^2(\mathbb{R}_k)}{20}\right]^2}{20}}} \end{cases} \tag{61}$$

where we use the $\mathbb{R}_i$ (i = 1, 2, . . . , 20) to represent the 20 native amino acids. The symbols h_0^1 and h_0^2 represent the original hydrophobicity and hydrophilicity values of the amino acid in the brackets right after the symbols.

For more information of the SC-PseAAC, please refer to (17).

3.3.3 General parallel correlation pseudo amino acid composition (PC-PseAAC-General)

The PC-PseAAC-General approach (14) cannot only incorporate comprehensive built-in indices (**Table 5**) extracted from AAindex (15), but also allow the users to upload their own indices to generate the PC-PseAAC-General feature vector.

Given a protein sequence **P** (**Eq.47**), the PC-PseAAC-General feature vector of **P** is defined:

$$\mathbf{P} = \begin{bmatrix} x_1 & x_2 & \cdots & x_{20} & x_{20+1} & \cdots & x_{20+\lambda} \end{bmatrix}^{\mathbf{T}} \tag{62}$$

where

$$x_u = \begin{cases} \dfrac{f_u}{\sum_{i=1}^{20} f_i + w\sum_{j=1}^{\lambda} \theta_j} & (1 \le u \le 20) \\ \dfrac{w\theta_{u-20}}{\sum_{i=1}^{20} f_i + w\sum_{j=1}^{\lambda} \theta_j} & (20+1 \le u \le 20+\lambda) \end{cases} \tag{63}$$

where f_i (i=1,2,···,20) is the normalized occurrence frequency of the 20 amino acids in the protein **P**; the parameter λ is an integer, representing the highest counted rank (or tier) of the correlation along a protein sequence; w is the weight factor ranging from 0 to 1; θ_j (j=1,2,···,λ) is called the j-tier correlation factor reflecting the sequence-order correlation between all the j-th most contiguous residues along a protein chain, which is defined:

$$\begin{cases} \theta_1 = \dfrac{1}{L\text{-}1}\sum_{i=1}^{L-1}\Theta(\mathrm{R}_i, \mathrm{R}_{i+1}) \\ \theta_2 = \dfrac{1}{L\text{-}2}\sum_{i=1}^{L-2}\Theta(\mathrm{R}_i, \mathrm{R}_{i+2}) \\ \theta_3 = \dfrac{1}{L\text{-}3}\sum_{i=1}^{L-3}\Theta(\mathrm{R}_i, \mathrm{R}_{i+3}) \quad (\lambda < L) \\ \cdots\cdots \\ \theta_\lambda = \dfrac{1}{L\text{-}\lambda}\sum_{i=1}^{L-\lambda}\Theta(\mathrm{R}_i, \mathrm{R}_{i+\lambda}) \end{cases} \tag{64}$$

where the correlation function is given by

$$\Theta(\mathrm{R}_i, \mathrm{R}_j) = \frac{1}{\mu}\sum_{u=1}^{\mu}[H_u(\mathrm{R}_i) - H_u(\mathrm{R}_j)]^2 \tag{65}$$

where μ is the number of physicochemical indices considered that listed in the **Table 5**; $H_u(\mathrm{R}_i)$ is the u-th physicochemical index value of the amino acid R_i; $H_u(\mathrm{R}_j)$, the u-th physicochemical index value for the amino acid R_j. Note that before substituting the physicochemical indices values into **Eq.65**, they are all subjected to a standard conversion as described by the following equation:

$$H_u(i) = \frac{H_u^0(i) - \sum_{i=1}^{20}\dfrac{H_u^0(i)}{20}}{\sqrt{\dfrac{\sum_{i=1}^{20}\left[H_u^0(i) - \sum_{i=1}^{20}\dfrac{H_u^0(i)}{20}\right]^2}{20}}} \tag{66}$$

where $H_u^0(i)$ is the u-th original physicochemical value of the i-th amino acid.

3.3.4 General series correlation pseudo amino acid composition (SC-PseAAC-General)

The SC-PseAAC-General approach (14) cannot only incorporate comprehensive built-in indices (**Table 5**) extracted from AAindex (15), but also allow the users to upload their own indices to generate the SC-PseAAC-General feature vector.

Given a protein sequence **P** (**Eq.47**), the SC-PseAAC-General feature vector of **P** is defined:

$$\mathbf{P} = \begin{bmatrix} p_1 & p_2 & \cdots & p_{20} & p_{20+1} & \cdots & p_{20+\lambda} & p_{20+\lambda+1} & \cdots & p_{20+\lambda\Lambda} \end{bmatrix}^{\mathbf{T}} \tag{67}$$

where

$$p_u = \begin{cases} \dfrac{f_u}{\sum_{i=1}^{20} f_i + w\sum_{j=1}^{\lambda\Lambda}\tau_j} & (1 \le u \le 20) \\ \dfrac{w\tau_{u-20}}{\sum_{i=1}^{20} f_i + w\sum_{j=1}^{\lambda\Lambda}\tau_j} & (20+1 \le u \le 20+\lambda\Lambda) \end{cases} \tag{68}$$

where f_i (i = 1, 2,... , 20) is the normalized occurrence frequency of the 20 native amino acids in the protein **P**, the parameter λ is an integer, representing the highest

counted rank (or tier) of the correlation along a protein sequence; w is the weight factor ranging from 0 to 1; Λ is the number of physicochemical indices (**Table 5**); τ_j the j -tier sequence-correlation factor reflecting the sequence-order correlation between all the most contiguous residues along a protein sequence, which is defined:

$$\begin{cases} \tau_1 = \dfrac{1}{L\text{-}1}\displaystyle\sum_{i=1}^{L-1} H_{i,i+1}^{1} \\ \tau_2 = \dfrac{1}{L\text{-}1}\displaystyle\sum_{i=1}^{L-1} H_{i,i+1}^{2} \\ \cdots\cdots \\ \tau_\Lambda = \dfrac{1}{L\text{-}1}\displaystyle\sum_{i=1}^{L-1} H_{i,i+1}^{\Lambda} \qquad \lambda < (L-1) \\ \cdots\cdots \\ \tau_{\lambda\Lambda-1} = \dfrac{1}{L\text{-}\lambda}\displaystyle\sum_{i=1}^{L-\lambda} H_{i,i+\lambda}^{\Lambda-1} \\ \tau_{\lambda\Lambda} = \dfrac{1}{L\text{-}\lambda}\displaystyle\sum_{i=1}^{L-\lambda} H_{i,i+\lambda}^{\Lambda} \end{cases} \tag{69}$$

where $H_{i,\mathrm{i+m}}^{\xi}$ is the correlation function given by

$$\begin{cases} H_{i,i+m}^{\zeta} = h^{\zeta}(\mathrm{R}_i)\cdot h^{\zeta}(\mathrm{R}_{i+m}) \\ \zeta = 1,2,\cdots,\Lambda;\ m=1,2,\cdots,\lambda;\ i=1,2,\cdots,L\text{-}m \end{cases} \tag{70}$$

where $h^{\zeta}(\mathrm{R}_i)$ is the ζ -th physicochemical value for the i-th (i = 1, 2, . . . ,L) amino acid in **Eq.47**, and the dot (•) means the multiplication sign.

Note that before substituting the physicochemical values into **Eq.70**, they are all subjected to a standard conversion as described by the following equation:

$$h^{\zeta}(\mathrm{R}_i) = \frac{h_0^{\zeta}(\mathrm{R}_i) - \displaystyle\sum_{k=1}^{20}\frac{h_0^{\zeta}(\mathbb{R}_k)}{20}}{\sqrt{\dfrac{\displaystyle\sum_{u=1}^{20}\left[h_0^{\zeta}(\mathbb{R}_u) - \displaystyle\sum_{k=1}^{20}\frac{h_0^{\zeta}(\mathbb{R}_k)}{20}\right]^2}{20}}} \tag{71}$$

where we use the $\mathbb{R}_i$ (i = 1, 2, . . . , 20) to represent the 20 native amino acids. The symbols h_0^{ζ} represent the ζ -th original physicochemical value of the amino acid in the brackets right after the symbols.

Table 1. The names of the 148 physicochemical indices for dinucleotides (DNA).

The values of 148 physicochemical indices can be found here.

Base stacking	Protein induced deformability	B-DNA twist
Propeller twist	Duplex stability:(freeenergy)	Duplex tability(disruptenergy)
Protein DNA twist	Stabilising energy of Z-DNA	Aida_BA_transition
Breslauer_dS	Electron_interaction	Hartman_trans_free_energy
Lisser_BZ_transition	Polar_interaction	SantaLucia_dG
Sarai_flexibility	Stability	Stacking_energy
Sugimoto_dS	Watson-Crick_interaction	Twist
Shift	Slide	Rise
Twist stiffness	Tilt stiffness	Shift_rise
Twist_shift	Enthalpy1	Twist_twist
Shift2	Tilt3	Tilt1
Slide (DNA-protein complex)1	Tilt_shift	Twist_tilt
Roll_rise	Stacking energy	Stacking energy1
Propeller Twist	Roll11	Rise (DNA-protein complex)
Roll2	Roll3	Roll1
Slide_slide	Enthalpy	Shift_shift
Flexibility_slide	Minor Groove Distance	Rise (DNA-protein complex)1
Roll (DNA-protein complex)1	Entropy	Cytosine content
Major Groove Distance	Twist (DNA-protein complex)	Purine (AG) content
Tilt_slide	Major Groove Width	Major Groove Depth
Free energy6	Free energy7	Free energy4
Free energy3	Free energy1	Twist_roll
Flexibility_shift	Shift (DNA-protein complex)1	Thymine content
Tip	Keto (GT) content	Roll stiffness
Entropy1	Roll_slide	Slide (DNA-protein complex)
Twist2	Twist5	Twist4
Tilt (DNA-protein complex)1	Twist_slide	Minor Groove Depth
Persistance Length	Rise3	Shift stiffness
Slide3	Slide2	Slide1
Rise1	Rise stiffness	Mobility to bend towards minor groove
Dinucleotide GC Content	A-philicity	Wedge
DNA denaturation	Bending stiffness	Free energy5
Breslauer_dG	Breslauer_dH	Shift (DNA-protein complex)
Helix-Coil_transition	Ivanov_BA_transition	Slide_rise
SantaLucia_dH	SantaLucia_dS	Minor Groove Width
Sugimoto_dG	Sugimoto_dH	Twist1
Tilt	Roll	Twist7
Clash Strength	Roll_roll	Roll (DNA-protein complex)

Adenine content	Direction	Probability contacting nucleosome core
Roll_shift	Shift_slide	Shift1
Tilt4	Tilt2	Free energy8
Twist (DNA-protein complex)1	Tilt_rise	Free energy2
Stacking energy2	Stacking energy3	Rise_rise
Tilt_tilt	Roll4	Tilt_roll
Minor Groove Size	GC content	Inclination
Slide stiffness	Melting Temperature1	Twist3
Tilt (DNA-protein complex)	Guanine content	Twist6
Major Groove Size	Twist_rise	Rise2
Melting Temperature	Free energy	Mobility to bend towards major groove
Bend		

Table 2. The names of the 12 physicochemical indices for trinucleotides (DNA).

For more information of the indices listed in this table, please click here, their values can be found here.

Bendability (DNAse)	Bendability (consensus)	Trinucleotide GC Content
Consensus_roll	Consensus-Rigid	Dnase I
MW-Daltons	MW-kg	Nucleosome
Nucleosome positioning	Dnase I-Rigid	Nucleosome-Rigid

Table 3. The names of the 6 physicochemical indices for dinucleotides (DNA).

For more information of the indices listed in this table, please click here, their values can be found here.

Twist(DNA)	Tilt(DNA)	Roll(DNA)
Shift(DNA)	Slide(DNA)	Rise(DNA)

Table 4. The names of the 22 physicochemical indices for dinucleotides (RNA).

For more information of the indices listed in this table, please click here, their values can be found here.

Shift (RNA)	Hydrophilicity (RNA)
Hydrophilicity (RNA)	GC content
Purine (AG) content	Keto (GT) content
Adenine content	Guanine content
Cytosine content	Thymine content
Slide (RNA)	Rise (RNA)
Tilt (RNA)	Roll (RNA)
Twist (RNA)	Stacking energy (RNA)
Enthalpy (RNA)	Entropy (RNA)
Free energy (RNA)	Free energy (RNA)
Enthalpy (RNA)	Entropy (RNA)

Table 5. The names of the 547 physicochemical indices for amino acids.

The meanings of the physicochemical indices can be found here, and their values can be found here .

Hydrophobicity	Hydrophilicity	Mass
ARGP820102	ARGP820103	BEGF750101
BHAR880101	BIGC670101	BIOV880101
BROC820102	BULH740101	BULH740102
BUNA790103	BURA740101	BURA740102
CHAM820102	CHAM830101	CHAM830102
CHAM830105	CHAM830106	CHAM830107
CHOC760101	CHOC760102	CHOC760103
CHOP780201	CHOP780202	CHOP780203
CHOP780206	CHOP780207	CHOP780208
CHOP780211	CHOP780212	CHOP780213
CHOP780216	CIDH920101	CIDH920102
CIDH920105	COHE430101	CRAJ730101
DAWD720101	DAYM780101	DAYM780201
EISD840101	EISD860101	EISD860102
FASG760102	FASG760103	FASG760104
FAUJ880101	FAUJ880102	FAUJ880103
FAUJ880106	FAUJ880107	FAUJ880108
FAUJ880111	FAUJ880112	FAUJ880113
FINA910102	FINA910103	FINA910104
GEIM800102	GEIM800103	GEIM800104
GEIM800107	GEIM800108	GEIM800109
GOLD730101	GOLD730102	GRAR740101
GUYH850101	HOPA770101	HOPT810101
HUTJ700103	ISOY800101	ISOY800102
ISOY800105	ISOY800106	ISOY800107
JANJ780102	JANJ780103	JANJ790101
JOND750102	JOND920101	JOND920102
KANM800101	KANM800102	KANM800103
KARP850102	KARP850103	KHAG800101
KRIW790101	KRIW790102	KRIW790103
LEVM760101	LEVM760102	LEVM760103
LEVM760106	LEVM760107	LEVM780101
LEVM780104	LEVM780105	LEVM780106
LIFS790102	LIFS790103	MANP780101
MAXF760103	MAXF760104	MAXF760105
MEEJ800101	MEEJ800102	MEEJ810101
MEIH800102	MEIH800103	MIYS850101
NAGK730103	NAKH900101	NAKH900102
NAKH900105	NAKH900106	NAKH900107
NAKH900110	NAKH900111	NAKH900112
NAKH920102	NAKH920103	NAKH920104
NAKH920107	NAKH920108	NISK800101
OOBM770101	OOBM770102	OOBM770103

OOBM850101	OOBM850102	OOBM850103
PALJ810101	PALJ810102	PALJ810103
PALJ810106	PALJ810107	PALJ810108
PALJ810111	PALJ810112	PALJ810113
PALJ810116	PARJ860101	PLIV810101
PONP800103	PONP800104	PONP800105
PONP800108	PRAM820101	PRAM820102
PRAM900102	PRAM900103	PRAM900104
QIAN880101	QIAN880102	QIAN880103
QIAN880106	QIAN880107	QIAN880108
QIAN880111	QIAN880112	QIAN880113
QIAN880116	QIAN880117	QIAN880118
QIAN880121	QIAN880122	QIAN880123
QIAN880126	QIAN880127	QIAN880128
QIAN880131	QIAN880132	QIAN880133
QIAN880136	QIAN880137	QIAN880138
RACS770102	RACS770103	RACS820101
RACS820104	RACS820105	RACS820106
RACS820109	RACS820110	RACS820111
RACS820114	RADA880101	RADA880102
RADA880105	RADA880106	RADA880107
RICJ880102	RICJ880103	RICJ880104
RICJ880107	RICJ880108	RICJ880109
RICJ880112	RICJ880113	RICJ880114
RICJ880117	ROBB760101	ROBB760102
ROBB760105	ROBB760106	ROBB760107
ROBB760110	ROBB760111	ROBB760112
ROSG850101	ROSG850102	ROSM880101
SIMZ760101	SNEP660101	SNEP660102
SUEM840101	SUEM840102	SWER830101
TANS770103	TANS770104	TANS770105
TANS770108	TANS770109	TANS770110
VASM830103	VELV850101	VENT840101
WEBA780101	WERD780101	WERD780102
WOEC730101	WOLR810101	WOLS870101
YUTK870101	YUTK870102	YUTK870103
ZIMJ680101	ZIMJ680102	ZIMJ680103
AURR980101	AURR980102	AURR980103
AURR980106	AURR980107	AURR980108
AURR980111	AURR980112	AURR980113
AURR980116	AURR980117	AURR980118
ONEK900101	ONEK900102	VINM940101
VINM940104	MUNV940101	MUNV940102
MUNV940105	WIMW960101	KIMC930101
PARS000101	PARS000102	KUMS000101
KUMS000104	TAKK010101	FODM020101
NADH010103	NADH010104	NADH010105
MONM990201	KOEP990101	KOEP990102
CEDJ970103	CEDJ970104	CEDJ970105

FUKS010103	FUKS010104	FUKS010105
FUKS010108	FUKS010109	FUKS010110
AVBF000101	AVBF000102	AVBF000103
AVBF000106	AVBF000107	AVBF000108
MITS020101	TSAJ990101	TSAJ990102
WILM950101	WILM950102	WILM950103
GUOD860101	JURD980101	BASU050101
SUYM030101	PUNT030101	PUNT030102
GEOR030103	GEOR030104	GEOR030105
GEOR030108	GEOR030109	ZHOH040101
BAEK050101	HARY940101	PONJ960101
OLSK800101	KIDA850101	GUYH850102
GUYH850105	ROSM880104	ROSM880105
BLAS910101	CASG920101	CORJ870101
CORJ870104	CORJ870105	CORJ870106
MIYS990101	MIYS990102	MIYS990103
ENGD860101	FASG890101	TANS770101
ANDN920101	ARGP820101	TANS770106
BEGF750102	BEGF750103	VASM830101
BIOV880102	BROC820101	VHEG790101
BUNA790101	BUNA790102	WERD780103
CHAM810101	CHAM820101	WOLS870102
CHAM830103	CHAM830104	YUTK870104
CHAM830108	CHOC750101	ZIMJ680104
CHOC760104	CHOP780101	AURR980104
CHOP780204	CHOP780205	AURR980109
CHOP780209	CHOP780210	AURR980114
CHOP780214	CHOP780215	AURR980119
CIDH920103	CIDH920104	VINM940102
CRAJ730102	CRAJ730103	MUNV940103
DESM900101	DESM900102	MONM990101
EISD860103	FASG760101	KUMS000102
FASG760105	FAUJ830101	NADH010101
FAUJ880104	FAUJ880105	NADH010106
FAUJ880109	FAUJ880110	CEDJ970101
FINA770101	FINA910101	FUKS010101
GARJ730101	GEIM800101	FUKS010106
GEIM800105	GEIM800106	FUKS010111
GEIM800110	GEIM800111	AVBF000104
GRAR740102	GRAR740103	AVBF000109
HUTJ700101	HUTJ700102	COSI940101
ISOY800103	ISOY800104	WILM950104
ISOY800108	JANJ780101	BASU050102
JANJ790102	JOND750101	GEOR030101
JUKT750101	JUNJ780101	GEOR030106
KANM800104	KARP850101	ZHOH040102
KLEP840101	KRIW710101	DIGM050101
KYTJ820101	LAWE840101	GUYH850103
LEVM760104	LEVM760105	JACR890101

LEVM780102	LEVM780103	CORJ870102
LEWP710101	LIFS790101	CORJ870107
MAXF760101	MAXF760102	MIYS990104
MAXF760106	MCMT640101	TANS770102
MEEJ810102	MEIH800101	TANS770107
NAGK730101	NAGK730102	VASM830102
NAKH900103	NAKH900104	WARP780101
NAKH900108	NAKH900109	WERD780104
NAKH900113	NAKH920101	WOLS870103
NAKH920105	NAKH920106	ZASB820101
NISK860101	NOZY710101	ZIMJ680105
OOBM770104	OOBM770105	AURR980105
OOBM850104	OOBM850105	AURR980110
PALJ810104	PALJ810105	AURR980115
PALJ810109	PALJ810110	AURR980120
PALJ810114	PALJ810115	VINM940103
PONP800101	PONP800102	MUNV940104
PONP800106	PONP800107	BLAM930101
PRAM820103	PRAM900101	KUMS000103
PTIO830101	PTIO830102	NADH010102
QIAN880104	QIAN880105	NADH010107
QIAN880109	QIAN880110	CEDJ970102
QIAN880114	QIAN880115	FUKS010102
QIAN880119	QIAN880120	FUKS010107
QIAN880124	QIAN880125	FUKS010112
QIAN880129	QIAN880130	AVBF000105
QIAN880134	QIAN880135	YANJ020101
QIAN880139	RACS770101	PONP930101
RACS820102	RACS820103	KUHL950101
RACS820107	RACS820108	BASU050103
RACS820112	RACS820113	GEOR030102
RADA880103	RADA880104	GEOR030107
RADA880108	RICJ880101	ZHOH040103
RICJ880105	RICJ880106	WOLR790101
RICJ880110	RICJ880111	GUYH850104
RICJ880115	RICJ880116	COWR900101
ROBB760103	ROBB760104	CORJ870103
ROBB760108	ROBB760109	CORJ870108
ROBB760113	ROBB790101	MIYS990105
ROSM880102	ROSM880103	SNEP660104
SNEP660103		

Table 6. The names of the 3 physicochemical indices for amino acids.

The values of 3 physicochemical indices can be found here.

Hydrophobicity	hydrophilicity	mass

Table 7. The names of the 2 physicochemical indices for amino acids.

The values of 2 physicochemical indices can be found here.

hydrophobicity	hydrophilicity

References

1. Lee, D., Karchin, R. and Beer, M.A. (2011) Discriminative prediction of mammalian enhancers from DNA sequence. *Genome Research*, **21**, 2167-2180.
2. Noble, W.S., Kuehn, S., Thurman, R., Yu, M. and Stamatoyannopoulos, J. (2005) Predicting the in vivo signature of human gene regulatory sequences. *Bioinformatics*, **21 Suppl 1**, i338-343.
3. Gupta, S., Dennis, J., Thurman, R.E., Kingston, R., Stamatoyannopoulos, J.A. and Noble, W.S. (2008) Predicting human nucleosome occupancy from primary sequence. *PLoS Computational Biology*, **4**, e1000134.
4. Dong, Q., Zhou, S. and Guan, J. (2009) A new taxonomy-based protein fold recognition approach based on autocross-covariance transformation. *Bioinformatics*, **25**, 2655-2662.
5. Guo, Y., Yu, L., Wen, Z. and Li, M. (2008) Using support vector machine combined with auto covariance to predict protein-protein interactions from protein sequences. *Nucleic Acids Res*, **36**, 3025-3030.
6. Chen, W., Zhang, X., Brooker, J., Lin, H., Zhang, L. and Chou, K.-C. (2014) PseKNC-General: a cross-platform package for generating various modes of pseudo nucleotide compositions. *Bioinformatics*, DOI: 10.1093/bioinformatics/btu1602.
7. Friedel, M., Nikolajewa, S., Suhnel, J. and Wilhelm, T. (2008) DiProDB: a database for dinucleotide properties. *Nucleic Acids Res*, **37**, D37-D40.
8. Chen, W., Feng, P.M., Lin, H. and Chou, K.C. (2013) iRSpot-PseDNC: identify recombination spots with pseudo dinucleotide composition. *Nucleic Acids Res*, **41**, e68.
9. Guo, S.H., Deng, E.Z., Xu, L.Q., Ding, H., Lin, H., Chen, W. and Chou, K.C. (2014) iNuc-PseKNC: a sequence-based predictor for predicting nucleosome positioning in genomes with pseudo k-tuple nucleotide composition. *Bioinformatics*, **30**, 1522-1529.
10. Lin, H., Deng, E.-Z., Ding, H., Chen, W. and Chou, K.-C. (2014) iPro54-PseKNC: a sequence-based predictor for identifying sigma-54 promoters in prokaryote with pseudo k-tuple nucleotide composition. *Nucleic Acids Res*, **42**, 12961-12972.
11. Chen, W., Lei, T.Y., Jin, D.C., Lin, H. and Chou, K.C. (2014) PseKNC: a flexible web server for generating pseudo K-tuple nucleotide composition. *Analytical biochemistry*, **456**, 53-60.
12. Wei, L., Liao, M., Gao, Y., Ji, R., He, Z. and Zou, Q. (2014) Improved and Promising Identification of Human MicroRNAs by Incorporating a High-quality Negative Set. *IEEE/ACM Transactions on Computational Biology and Bioinformatics*, **11**, 192-201.
13. Liu, B., Wang, X., Lin, L., Dong, Q. and Wang, X. (2008) A Discriminative Method for Protein Remote Homology Detection and Fold Recognition Combining Top-n-grams and Latent Semantic Analysis. *BMC Bioinformatics*, **9**, 510.
14. Cao, D.-S., Xu, Q.-S. and Liang, Y.-Z. (2013) propy: a tool to generate various modes of Chou's PseAAC. *Bioinformatics*, **29**, 960-962.
15. Kawashima, S., Pokarowski, P., Pokarowska, M., Kolinski, A., Katayama, T. and Kanehisa, M. (2008) AAindex: amino acid index database, progress report 2008. *Nucleic Acids Res*, **36**, D202-D205.
16. Chou, K.-C. (2001) Prediction of protein cellular attributes using pseudo-amino-acid-composition. *PROTEINS: Structure, Function, and Genetics*, **43**, 246-255.
17. Chou, K.-C. (2005) Using amphiphilic pseudo amino acid composition to predict enzyme subfamily classes. *Bioinformatics*, **21**, 10-19.
