# Supplementary material for: Pse-in-One: a web server for generating various modes of pseudo components of DNA, RNA, and protein sequences
Source: Nucleic Acids Res. 2015 May 9;43(Web Server issue):W65–71. doi: 10.1093/nar/gkv458 (PMC4489303; doi:10.1093/nar/gkv458)
Supplement: SUPPLEMENTARY DATA [file supp_gkv458_nar-00118-web-b-2015-File003.pdf]

Supplementary Description S2. Usage of Pse-in-One

Listed here are a series of publications in which the key approach is in using the pseudo k-tuple nucleotide composition or pseudo amino acid composition, as reflected by the fact that the titles of all these papers explicitly contain the terms "PseKNC", "pseudo nucleotide composition", "PseAAC", or "pseudo amino acid composition" (1-194).

For these ongoing studies or topics in computational biology, it would be much more efficient by using **Pse-in-One.** Particularly, many of these topics are vitally important for both basic research and drug development, such as identification of recombination spots (22,155), identification of nucleosome positioning (1,195), prediction of promoters (2,173), identification of translation initiation sites (174), prediction of methylation status and sites (134,194,196), detecting splicing sites (175), identifying DNase I hypersensitive sites (177), identifying microRNA precursors (192,193), predicting posttranslational modification (PTM) sites in proteins (16,17,23-25,179,185,197), detecting protein remote homology (166), predicting antifreeze proteins (182), predicting protein structural class (180,187), predicting anticancer peptides (178), identifying bacterial virulent proteins (147), predicting protein subcellular location in various organisms and levels (10,21,26,28,75,82,93,102,188), predicting membrane protein types (19,156), discriminating outer membrane proteins (7,73), predicting beta-lactamase (191), discrimination of acidic and alkaline enzyme (190), analyzing genetic sequence (159), identifying cyclin proteins (104), predicting GABA(A) receptor proteins (124), identifying antibacterial peptides (164), identifying allergenic proteins (167), predicting metalloproteinase family (125), identifying GPCRs and their types (154), identifying protein quaternary structural attributes (12), identifying risk type of human papillomaviruses (98), and predicting drug-target interaction in cellular networking (14,198-202).

REFERENCES

1. Guo, S.H., Deng, E.Z., Xu, L.Q., Ding, H., Lin, H., Chen, W. and Chou, K.C. (2014) iNuc-PseKNC: a sequence-based predictor for predicting nucleosome positioning in genomes with pseudo k-tuple nucleotide composition. *Bioinformatics*, **30**, 1522-1529.
2. Lin, H., Deng, E.Z., Ding, H., Chen, W. and Chou, K.C. (2014) iPro54-PseKNC: a sequence-based predictor for identifying sigma-54 promoters in prokaryote with pseudo k-tuple nucleotide composition. *Nucleic Acids Res.*, **42**, 12961-12972.
3. Zhang, S.W., Chen, W., Yang, F. and Pan, Q. (2008) Using Chou's pseudo amino acid composition to predict protein quaternary structure: a sequence-segmented PseAAC approach. *Amino Acids*, **35**, 591-598.
4. Nanni, L., Brahnam, S. and Lumini, A. (2010) High performance set of PseAAC and sequence based descriptors for protein classification. *J. Theor. Biol.*, **266**, 1-10.
5. Liao, B., Jiang, J.B., Zeng, Q.G. and Zhu, W. (2011) Predicting Apoptosis Protein

Subcellular Location with PseAAC by Incorporating Tripeptide Composition. *Protein & Peptide Letters*, **18**, 1086-1092.
6. Du, P., Wang, X., Xu, C. and Gao, Y. (2012) PseAAC-Builder: A cross-platform stand-alone program for generating various special Chou's pseudo-amino acid compositions. *Anal. Biochem.*, **425**, 117-119.
7. Hayat, M. and Khan, A. (2012) Discriminating Outer Membrane Proteins with Fuzzy K-Nearest Neighbor Algorithms Based on the General Form of Chou's PseAAC. *Protein & Peptide Letters*, **19**, 411-421.
8. Liao, B., Xiang, Q. and Li, D. (2012) Incorporating Secondary Features into the General form of Chou's PseAAC for Predicting Protein Structural Class. *Protein & Peptide Letters*, **19**, 1133-1138.
9. Mei, S. (2012) Multi-kernel transfer learning based on Chou's PseAAC formulation for protein submitochondria localization. *J. Theor. Biol.*, **293**, 121-130.
10. Mei, S. (2012) Predicting plant protein subcellular multi-localization by Chou's PseAAC formulation based multi-label homolog knowledge transfer learning. *J. Theor. Biol.*, **310**, 80-87.
11. Qin, Y.F., Wang, C.H., Yu, X.Q., Zhu, J., Liu, T.G. and Zheng, X.Q. (2012) Predicting Protein Structural Class by Incorporating Patterns of Over-Represented k-mers into the General form of Chou's PseAAC. *Protein & Peptide Letters*, **19**, 388-397.
12. Sun, X.Y., Shi, S.P., Qiu, J.D., Suo, S.B., Huang, S.Y. and Liang, R.P. (2012) Identifying protein quaternary structural attributes by incorporating physicochemical properties into the general form of Chou's PseAAC via discrete wavelet transform. *Molecular BioSystems*, **8**, 3178-3184.
13. Chang, T.H., Wu, L.C., Lee, T.Y., Chen, S.P., Huang, H.D. and Horng, J.T. (2013) EuLoc: a web-server for accurately predict protein subcellular localization in eukaryotes by incorporating various features of sequence segments into the general form of Chou's PseAAC. *Journal of Computer-Aided Molecular Design*, **27**, 91-103.
14. Xiao, X., Min, J.L., Wang, P. and Chou, K.C. (2013) iCDI-PseFpt: Identify the channel-drug interaction in cellular networking with PseAAC and molecular fingerprints. *J. Theor. Biol.*, **337C**, 71-79.
15. Xie, H.L., Fu, L. and Nie, X.D. (2013) Using ensemble SVM to identify human GPCRs N-linked glycosylation sites based on the general form of Chou's PseAAC. *Protein Eng Des Sel*, **26**, 735-742.
16. Xu, Y., Ding, J., Wu, L.Y. and Chou, K.C. (2013) iSNO-PseAAC: Predict cysteine S-nitrosylation sites in proteins by incorporating position specific amino acid propensity into pseudo amino acid composition *PLoS ONE*, **8**, e55844.
17. Xu, Y., Shao, X.J., Wu, L.Y., Deng, N.Y. and Chou, K.C. (2013) iSNO-AAPair: incorporating amino acid pairwise coupling into PseAAC for predicting cysteine S-nitrosylation sites in proteins. *PeerJ*, **1**, e171.
18. Du, P., Gu, S. and Jiao, Y. (2014) PseAAC-General: Fast building various modes of general form of Chou's pseudo-amino acid composition for large-scale protein datasets. *International Journal of Molecular Sciences*, **15**, 3495-3506.
19. Han, G.S., Yu, Z.G. and Anh, V. (2014) A two-stage SVM method to predict

membrane protein types by incorporating amino acid classifications and physicochemical properties into a general form of Chou's PseAAC. *J. Theor. Biol.*, **344**, 31-39.
20. Hayat, M. and Iqbal, N. (2014) Discriminating protein structure classes by incorporating Pseudo Average Chemical Shift to Chou's general PseAAC and Support Vector Machine. *Computer methods and programs in biomedicine*, **116**, 184-192.
21. Li, L., Yu, S., Xiao, W., Li, Y., Li, M., Huang, L., Zheng, X., Zhou, S. and Yang, H. (2014) Prediction of bacterial protein subcellular localization by incorporating various features into Chou's PseAAC and a backward feature selection approach. *Biochimie*, **104**, 100-107.
22. Qiu, W.R., Xiao, X. and Chou, K.C. (2014) iRSpot-TNCPseAAC: Identify recombination spots with trinucleotide composition and pseudo amino acid components. *Int J Mol Sci*, **15**, 1746-1766.
23. Qiu, W.R., Xiao, X., Lin, W.Z. and Chou, K.C. (2014) iMethyl-PseAAC: Identification of Protein Methylation Sites via a Pseudo Amino Acid Composition Approach. *Biomed Res Int*, **2014**, 947416.
24. Xu, Y., Wen, X., Shao, X.J., Deng, N.Y. and Chou, K.C. (2014) iHyd-PseAAC: Predicting hydroxyproline and hydroxylysine in proteins by incorporating dipeptide position-specific propensity into pseudo amino acid composition. *Int. J. Mol. Sci.*, **15**, 7594-7610.
25. Zhang, J., Zhao, X., Sun, P. and Ma, Z. (2014) PSNO: Predicting Cysteine S-Nitrosylation Sites by Incorporating Various Sequence-Derived Features into the General Form of Chou's PseAAC. *Int J Mol Sci*, **15**, 11204-11219.
26. Dehzangi, A., Heffernan, R., Sharma, A., Lyons, J., Paliwal, K. and Sattar, A. (2015) Gram-positive and Gram-negative protein subcellular localization by incorporating evolutionary-based descriptors into Chou's general PseAAC. *Journal of Theoretical Biology*, **364**, 284-294.
27. Liu, B., Xu, J., Fan, S., Xu, R., Jiyun Zhou, J. and Wang, X. (2015) PseDNA-Pro: DNA-binding protein identification by combining Chou's PseAAC and physicochemical distance transformation. *Molecular Informatics*, **34**, 8-17
28. Mandal, M., Mukhopadhyay, A. and Maulik, U. (2015) Prediction of protein subcellular localization by incorporating multiobjective PSO-based feature subset selection into the general form of Chou's PseAAC. *Medical & biological engineering & computing*.
29. Chou, K.C. (2001) Prediction of protein cellular attributes using pseudo amino acid composition. *PROTEINS: Structure, Function, and Genetics (Erratum: ibid., 2001, Vol.44, 60)*, **43**, 246-255.
30. Guo, Z.M. (2002) Prediction of Membrane protein types by using pattern recognition method based on pseudo amino acid composition. *Master Thesis, Bio-X Life Science Research Center, Shanghai Jiaotong University*.
31. Haddadnia, J., Faez, K. and Ahmadi, M. (2002) A neural based human face recognition system using an efficient feature extraction method with pseudo zernike moment. *Journal of Circuits, Systems, and Computers*, **11**, 283-304.
32. Cai, Y.D. and Chou, K.C. (2003) Nearest neighbour algorithm for predicting protein subcellular location by combining functional domain composition and

pseudo-amino acid composition. *Biochem Biophys Res Comm (BBRC)*, **305**, 407-411.
33. Chou, K.C. and Cai, Y.D. (2003) Predicting protein quaternary structure by pseudo amino acid composition. *Proteins: Struct., Funct., Genet.*, **53**, 282-289.
34. Chou, K.C. and Cai, Y.D. (2003) Prediction and classification of protein subcellular location: sequence-order effect and pseudo amino acid composition. *Journal of Cellular Biochemistry (Addendum, ibid. 2004, 91, 1085)*, **90**, 1250-1260.
35. Pan, Y.X., Zhang, Z.Z., Guo, Z.M., Feng, G.Y., Huang, Z.D. and He, L. (2003) Application of pseudo amino acid composition for predicting protein subcellular location: stochastic signal processing approach. *J. Protein Chem.*, **22**, 395-402.
36. Chou, K.C. and Cai, Y.D. (2004) Predicting subcellular localization of proteins by hybridizing functional domain composition and pseudo-amino acid composition. *J. Cell. Biochem.*, **91**, 1197-1203.
37. Wang, M., Yang, J., Liu, G.P., Xu, Z.J. and Chou, K.C. (2004) Weighted-support vector machines for predicting membrane protein types based on pseudo amino acid composition. *Protein Engineering, Design, and Selection*, **17**, 509-516.
38. Cai, Y.D. and Chou, K.C. (2005) Predicting enzyme subclass by functional domain composition and pseudo amino acid composition. *Journal of Proteome Research*, **4**, 967-971.
39. Cai, Y.D., Zhou, G.P. and Chou, K.C. (2005) Predicting enzyme family classes by hybridizing gene product composition and pseudo-amino acid composition. *J. Theor. Biol.*, **234**, 145-149.
40. Chou, K.C. (2005) Using amphiphilic pseudo amino acid composition to predict enzyme subfamily classes. *Bioinformatics*, **21**, 10-19.
41. Gao, Y., Shao, S.H., Xiao, X., Ding, Y.S., Huang, Y.S., Huang, Z.D. and Chou, K.C. (2005) Using pseudo amino acid composition to predict protein subcellular location: approached with Lyapunov index, Bessel function, and Chebyshev filter. *Amino Acids*, **28**, 373-376.
42. Liu, H., Yang, J., Wang, M., Xue, L. and Chou, K.C. (2005) Using Fourier spectrum analysis and pseudo amino acid composition for prediction of membrane protein types. *The Protein Journal*, **24**, 385-389.
43. Shen, H.B. and Chou, K.C. (2005) Using optimized evidence-theoretic K-nearest neighbor classifier and pseudo amino acid composition to predict membrane protein types. *Biochemical & Biophysical Research Communications (BBRC)*, **334**, 288-292.
44. Shen, H.B. and Chou, K.C. (2005) Predicting protein subnuclear location with optimized evidence-theoretic K-nearest classifier and pseudo amino acid composition. *Biochem Biophys Res Comm. (BBRC)*, **337**, 752-756.
45. Xue, C., Li, F., He, T., Liu, G.P., Li, Y. and Zhang, X. (2005) Classification of real and pseudo microRNA precursors using local structure-sequence features and support vector machine. *BMC Bioinformatics*, **6**, 310.
46. Cai, Y.D. and Chou, K.C. (2006) Predicting membrane protein type by functional domain composition and pseudo amino acid composition. *J. Theor.*

Biol., **238**, 395-400.
47. Chen, C., Tian, Y.X., Zou, X.Y., Cai, P.X. and Mo, J.Y. (2006) Using pseudo-amino acid composition and support vector machine to predict protein structural class. *J. Theor. Biol.*, **243**, 444-448.
48. Chen, C., Zhou, X., Tian, Y., Zou, X. and Cai, P. (2006) Predicting protein structural class with pseudo-amino acid composition and support vector machine fusion network. *Anal. Biochem.*, **357**, 116-121.
49. Du, P. and Li, Y. (2006) Prediction of protein submitochondria locations by hybridizing pseudo-amino acid composition with various physicochemical features of segmented sequence. *BMC Bioinformatics*, **7**, 518.
50. Mondal, S., Bhavna, R., Mohan Babu, R. and Ramakumar, S. (2006) Pseudo amino acid composition and multi-class support vector machines approach for conotoxin superfamily classification. *J. Theor. Biol.*, **243**, 252-260.
51. Shen, H.B., Yang, J. and Chou, K.C. (2006) Fuzzy KNN for predicting membrane protein types from pseudo amino acid composition. *J. Theor. Biol.*, **240**, 9-13.
52. Wang, S.Q., Yang, J. and Chou, K.C. (2006) Using stacked generalization to predict membrane protein types based on pseudo amino acid composition. *J. Theor. Biol.*, **242**, 941-946.
53. Xiao, X., Shao, S.H., Ding, Y.S., Huang, Z.D. and Chou, K.C. (2006) Using cellular automata images and pseudo amino acid composition to predict protein subcellular location. *Amino Acids*, **30**, 49-54.
54. Xiao, X., Shao, S.H., Huang, Z.D. and Chou, K.C. (2006) Using pseudo amino acid composition to predict protein structural classes: approached with complexity measure factor. *J. Comput. Chem.*, **27**, 478-482.
55. Zhang, S.W., Pan, Q., Zhang, H.C., Shao, Z.C. and Shi, J.Y. (2006) Prediction protein homo-oligomer types by pseudo amino acid composition: Approached with an improved feature extraction and naive Bayes feature fusion. *Amino Acids*, **30**, 461-468.
56. Zhou, G.P. and Cai, Y.D. (2006) Predicting protease types by hybridizing gene ontology and pseudo amino acid composition. *PROTEINS: Structure, Function, and Bioinformatics*, **63**, 681-684.
57. Chen, Y.L. and Li, Q.Z. (2007) Prediction of apoptosis protein subcellular location using improved hybrid approach and pseudo amino acid composition. *J. Theor. Biol.*, **248**, 377–381.
58. Ding, Y.S., Zhang, T.L. and Chou, K.C. (2007) Prediction of protein structure classes with pseudo amino acid composition and fuzzy support vector machine network. *Protein & Peptide Letters*, **14**, 811-815.
59. Lin, H. and Li, Q.Z. (2007) Predicting conotoxin superfamily and family by using pseudo amino acid composition and modified Mahalanobis discriminant. *Biochem. Biophys. Res. Commun.*, **354**, 548-551.
60. Lin, H. and Li, Q.Z. (2007) Using Pseudo Amino Acid Composition to Predict Protein Structural Class: Approached by Incorporating 400 Dipeptide Components. *Journal of Computational Chemistry*, **28**, 1463-1466.
61. Mundra, P., Kumar, M., Kumar, K.K., Jayaraman, V.K. and Kulkarni, B.D. (2007) Using pseudo amino acid composition to predict protein subnuclear localization: Approached with PSSM. *Pattern Recognition Letters*, **28**, 1610-

1615.
62. Oxenoid, K., Rice, A.J. and Chou, J.J. (2007) Comparing the structure and dynamics of phospholamban pentamer in its unphosphorylated and pseudo-phosphorylated states. *Protein Sci*, **16**, 1977-1983.
63. Shi, J.Y., Zhang, S.W., Pan, Q., Cheng, Y.-M. and Xie, J. (2007) Prediction of protein subcellular localization by support vector machines using multi-scale energy and pseudo amino acid composition. *Amino Acids*, **33**, 69-74.
64. Zhang, T.L. and Ding, Y.S. (2007) Using pseudo amino acid composition and binary-tree support vector machines to predict protein structural classes. *Amino Acids*, **33**, 623-629.
65. Zhou, X.B., Chen, C., Li, Z.C. and Zou, X.Y. (2007) Using Chou's amphiphilic pseudo-amino acid composition and support vector machine for prediction of enzyme subfamily classes. *J. Theor. Biol.*, **248**, 546–551.
66. Diao, Y., Ma, D., Wen, Z., Yin, J., Xiang, J. and Li, M. (2008) Using pseudo amino acid composition to predict transmembrane regions in protein: cellular automata and Lempel-Ziv complexity. *Amino Acids*, **34**, 111-117.
67. Ding, Y.S. and Zhang, T.L. (2008) Using Chou's pseudo amino acid composition to predict subcellular localization of apoptosis proteins: an approach with immune genetic algorithm-based ensemble classifier. *Pattern Recognition Letters*, **29**, 1887-1892.
68. Fang, Y., Guo, Y., Feng, Y. and Li, M. (2008) Predicting DNA-binding proteins: approached from Chou's pseudo amino acid composition and other specific sequence features. *Amino Acids*, **34**, 103-109.
69. Jiang, X., Wei, R., Zhang, T.L. and Gu, Q. (2008) Using the concept of Chou's pseudo amino acid composition to predict apoptosis proteins subcellular location: an approach by approximate entropy. *Protein & Peptide Letters*, **15**, 392-396.
70. Jiang, X., Wei, R., Zhao, Y. and Zhang, T. (2008) Using Chou's pseudo amino acid composition based on approximate entropy and an ensemble of AdaBoost classifiers to predict protein subnuclear location. *Amino Acids*, **34**, 669-675.
71. Li, F.M. and Li, Q.Z. (2008) Using pseudo amino acid composition to predict protein subnuclear location with improved hybrid approach. *Amino Acids*, **34**, 119-125.
72. Li, F.M. and Li, Q.Z. (2008) Predicting protein subcellular location using Chou's pseudo amino acid composition and improved hybrid approach. *Protein & Peptide Letters*, **15**, 612-616.
73. Lin, H. (2008) The modified Mahalanobis discriminant for predicting outer membrane proteins by using Chou's pseudo amino acid composition. *J. Theor. Biol.*, **252**, 350-356.
74. Lin, H., Ding, H., Feng-Biao Guo, F.B., Zhang, A.Y. and Huang, J. (2008) Predicting subcellular localization of mycobacterial proteins by using Chou's pseudo amino acid composition. *Protein & Peptide Letters*, **15**, 739-744.
75. Nanni, L. and Lumini, A. (2008) Genetic programming for creating Chou's pseudo amino acid based features for submitochondria localization. *Amino Acids*, **34**, 653-660.

76. Shen, H.B. and Chou, K.C. (2008) PseAAC: a flexible web-server for generating various kinds of protein pseudo amino acid composition. *Anal. Biochem.*, **373**, 386-388.
77. Shi, J.Y., Zhang, S.W., Pan, Q. and Zhou, G.P. (2008) Using Pseudo Amino Acid Composition to Predict Protein Subcellular Location: Approached with Amino Acid Composition Distribution. *Amino Acids*, **35**, 321-327.
78. Xiao, X., Lin, W.Z. and Chou, K.C. (2008) Using grey dynamic modeling and pseudo amino acid composition to predict protein structural classes. *J. Comput. Chem.*, **29**, 2018-2024.
79. Xiao, X., Wang, P. and Chou, K.C. (2008) Predicting protein structural classes with pseudo amino acid composition: an approach using geometric moments of cellular automaton image. *J. Theor. Biol.*, **254**, 691-696.
80. Zhang, G.Y. and Fang, B.S. (2008) Predicting the cofactors of oxidoreductases based on amino acid composition distribution and Chou's amphiphilic pseudo amino acid composition. *J. Theor. Biol.*, **253**, 310-315.
81. Zhang, G.Y., Li, H.C., Gao, J.Q. and Fang, B.S. (2008) Predicting lipase types by improved Chou's pseudo-amino acid composition. *Protein & Peptide Letters*, **15**, 1132-1137.
82. Zhang, S.W., Zhang, Y.L., Yang, H.F., Zhao, C.H. and Pan, Q. (2008) Using the concept of Chou's pseudo amino acid composition to predict protein subcellular localization: an approach by incorporating evolutionary information and von Neumann entropies. *Amino Acids*, **34**, 565-572.
83. Zhang, T.L., Ding, Y.S. and Chou, K.C. (2008) Prediction protein structural classes with pseudo amino acid composition: approximate entropy and hydrophobicity pattern. *J. Theor. Biol.*, **250**, 186-193.
84. Aguero-Chapin, G., Varona-Santos, J., de la Riva, G.A., Antunes, A., Gonzalez-Villa, T., Uriarte, E. and Gonzalez-Diaz, H. (2009) Alignment-Free Prediction of Polygalacturonases with Pseudofolding Topological Indices: Experimental Isolation from Coffea arabica and Prediction of a New Sequence. *Journal of Proteome Research*, **8**, 2122-2128.
85. Aguero-Chapin, G., Varona-Santos, J., de la Riva, G.A., Antunes, A., Gonzalez-Vlla, T., Uriarte, E. and Gonzalez-Diaz, H. (2009) Alignment-free prediction of polygalacturonases with pseudofolding topological indices: experimental isolation from Coffea arabica and prediction of a new sequence. *J Proteome Res*, **8**, 2122-2128.
86. Chen, C., Chen, L., Zou, X. and Cai, P. (2009) Prediction of protein secondary structure content by using the concept of Chou's pseudo amino acid composition and support vector machine. *Protein & Peptide Letters*, **16**, 27-31.
87. Chou, K.C. (2009) Pseudo amino acid composition and its applications in bioinformatics, proteomics and system biology. *Current Proteomics*, **6**, 262-274.
88. Ding, H., Luo, L. and Lin, H. (2009) Prediction of cell wall lytic enzymes using Chou's amphiphilic pseudo amino acid composition. *Protein & Peptide Letters*, **16**, 351-355.
89. Du, P., Cao, S. and Li, Y. (2009) SubChlo: predicting protein subchloroplast locations with pseudo-amino acid composition and the evidence-theoretic K-

nearest neighbor (ET-KNN) algorithm. *Journal of Theoretical Biolology*, **261**, 330-335.

90. Gao, Q.B., Jin, Z.C., Ye, X.F., Wu, C. and He, J. (2009) Prediction of nuclear receptors with optimal pseudo amino acid composition. *Anal. Biochem.*, **387**, 54-59.
91. Georgiou, D.N., Karakasidis, T.E., Nieto, J.J. and Torres, A. (2009) Use of fuzzy clustering technique and matrices to classify amino acids and its impact to Chou's pseudo amino acid composition. *J. Theor. Biol.*, **257**, 17-26.
92. Li, Z.C., Zhou, X.B., Dai, Z. and Zou, X.Y. (2009) Prediction of protein structural classes by Chou's pseudo amino acid composition: approached using continuous wavelet transform and principal component analysis. *Amino Acids*, **37**, 415-425.
93. Lin, H., Wang, H., Ding, H., Chen, Y.L. and Li, Q.Z. (2009) Prediction of Subcellular Localization of Apoptosis Protein Using Chou's Pseudo Amino Acid Composition. *Acta Biotheoretica*, **57**, 321-330.
94. Perez-Bello, A., Munteanu, C.R., Ubeira, F.M., De Magalhaes, A.L., Uriarte, E. and Gonzalez-Diaz, H. (2009) Alignment-free prediction of mycobacterial DNA promoters based on pseudo-folding lattice network or star-graph topological indices. *J. Theor. Biol.*, **256**, 458-466.
95. Qiu, J.D., Huang, J.H., Liang, R.P. and Lu, X.Q. (2009) Prediction of G-protein-coupled receptor classes based on the concept of Chou's pseudo amino acid composition: an approach from discrete wavelet transform. *Anal. Biochem.*, **390**, 68-73.
96. Xiao, X., Wang, P. and Chou, K.C. (2009) Predicting protein quaternary structural attribute by hybridizing functional domain composition and pseudo amino acid composition. *J. Appl. Crystallogr.*, **42**, 169-173.
97. Zeng, Y.H., Guo, Y.Z., Xiao, R.Q., Yang, L., Yu, L.Z. and Li, M.L. (2009) Using the augmented Chou's pseudo amino acid composition for predicting protein submitochondria locations based on auto covariance approach. *J. Theor. Biol.*, **259**, 366–372.
98. Esmaeili, M., Mohabatkar, H. and Mohsenzadeh, S. (2010) Using the concept of Chou's pseudo amino acid composition for risk type prediction of human papillomaviruses. *J. Theor. Biol.*, **263**, 203-209.
99. Gao, Q.B., Ye, X.F., Jin, Z.C. and He, J. (2010) Improving discrimination of outer membrane proteins by fusing different forms of pseudo amino acid composition. *Anal. Biochem.*, **398**, 52-59.
100. Gu, Q., Ding, Y., Zhang, T. and Shen, Y. (2010) Prediction of G-protein-coupled receptor classes with pseudo amino acid composition. *Sheng Wu Yi Xue Gong Cheng Xue Za Zhi*, **27**, 500-504.
101. Gu, Q., Ding, Y.S. and Zhang, T.L. (2010) Prediction of G-Protein-Coupled Receptor Classes in Low Homology Using Chou's Pseudo Amino Acid Composition with Approximate Entropy and Hydrophobicity Patterns. *Protein & Peptide Letters*, **17**, 559-567.
102. Kandaswamy, K.K., Pugalenthi, G., Moller, S., Hartmann, E., Kalies, K.U., Suganthan, P.N. and Martinetz, T. (2010) Prediction of Apoptosis Protein Locations with Genetic Algorithms and Support Vector Machines Through a

New Mode of Pseudo Amino Acid Composition. *Protein and Peptide Letters*, **17**, 1473-1479.
103. Liu, T., Zheng, X., Wang, C. and Wang, J. (2010) Prediction of Subcellular Location of Apoptosis Proteins using Pseudo Amino Acid Composition: An Approach from Auto Covariance Transformation. *Protein & Peptide Letters*, **17**, 1263-1269.
104. Mohabatkar, H. (2010) Prediction of cyclin proteins using Chou's pseudo amino acid composition. *Protein & Peptide Letters*, **17**, 1207-1214.
105. Niu, X.H., Li, N.N., Shi, F., Hu, X.H., Xia, J.B. and Xiong, H.J. (2010) Predicting protein solubility with a hybrid approach by pseudo amino Acid composition. *Protein and Peptide Letters*, **17**, 1466-1472.
106. Qiu, J.D., Huang, J.H., Shi, S.P. and Liang, R.P. (2010) Using the concept of Chou's pseudo amino acid composition to predict enzyme family classes: an approach with support vector machine based on discrete wavelet transform. *Protein & Peptide Letters*, **17**, 715-722.
107. Sahu, S.S. and Panda, G. (2010) A novel feature representation method based on Chou's pseudo amino acid composition for protein structural class prediction. *Computational Biology and Chemistry*, **34**, 320-327.
108. Wang, Y.C., Wang, X.B., Yang, Z.X. and Deng, N.Y. (2010) Prediction of enzyme subfamily class via pseudo amino acid composition by incorporating the conjoint triad feature. *Protein & Peptide Letters*, **17**, 1441-1449.
109. Wu, J., Li, M.L., Yu, L.Z. and Wang, C. (2010) An ensemble classifier of support vector machines used to predict protein structural classes by fusing auto covariance and pseudo-amino acid composition. *Protein J*, **29**, 62-67.
110. Yu, L., Guo, Y., Li, Y., Li, G., Li, M., Luo, J., Xiong, W. and Qin, W. (2010) SecretP: Identifying bacterial secreted proteins by fusing new features into Chou's pseudo-amino acid composition. *J. Theor. Biol.*, **267**, 1-6.
111. Chou, K.C. (2011) Some remarks on protein attribute prediction and pseudo amino acid composition (50th Anniversary Year Review). *J. Theor. Biol.*, **273**, 236-247.
112. Ding, H., Liu, L., Guo, F.B., Huang, J. and Lin, H. (2011) Identify Golgi protein types with modified mahalanobis discriminant algorithm and pseudo amino acid composition. *Protein & Peptide Letters*, **18**, 58-63.
113. Guo, J., Rao, N., Liu, G., Yang, Y. and Wang, G. (2011) Predicting protein folding rates using the concept of Chou's pseudo amino acid composition. *Journal of Computational Chemistry*, **32**, 1612-1617.
114. Hayat, M. and Khan, A. (2011) Predicting membrane protein types by fusing composite protein sequence features into pseudo amino acid composition. *J. Theor. Biol.*, **271**, 10-17.
115. Hu, L., Zheng, L., Wang, Z., Li, B. and Liu, L. (2011) Using pseudo amino Acid composition to predict protease families by incorporating a series of protein biological features. *Protein and Peptide Letters*, **18**, 552-558.
116. Huang, Y., Yang, L. and Wang, T. (2011) Phylogenetic analysis of DNA sequences based on the generalized pseudo-amino acid composition. *J. Theor. Biol.*, **269**, 217-223.
117. Huang, Y., Yang, L. and Wang, T. (2011) Phylogenetic analysis of DNA

sequences based on the generalized pseudo-amino acid composition. *J. Theor. Biol.*, **269**, 217-223.

118. Jingbo, X., Silan, Z., Feng, S., Huijuan, X., Xuehai, H., Xiaohui, N. and Zhi, L. (2011) Using the concept of pseudo amino acid composition to predict resistance gene against Xanthomonas oryzae pv. oryzae in rice: An approach from chaos games representation. *Journal of Theoretical Biology*, **284**, 16-23.
119. Lin, H. and Ding, H. (2011) Predicting ion channels and their types by the dipeptide mode of pseudo amino acid composition. *J. Theor. Biol.*, **269**, 64-69.
120. Lin, J. and Wang, Y. (2011) Using a novel AdaBoost algorithm and Chou's pseudo amino acid composition for predicting protein subcellular localization. *Protein & Peptide Letters*, **18**, 1219-1225.
121. Lin, J., Wang, Y. and Xu, X. (2011) A novel ensemble and composite approach for classifying proteins based on Chou's pseudo amino acid composition. *African Journal of Biotechnology*, **10**, 16963-16968.
122. Liu, X.L., Lu, J.L. and Hu, X.H. (2011) Predicting Thermophilic Proteins with Pseudo Amino Acid Composition: Approached from Chaos Game Representation and Principal Component Analysis. *Protein & Peptide Letters*, **18**, 1244-1250.
123. Mahdavi, A. and Jahandideh, S. (2011) Application of density similarities to predict membrane protein types based on pseudo-amino acid composition. *J. Theor. Biol.*, **276**, 132-137.
124. Mohabatkar, H., Mohammad Beigi, M. and Esmaeili, A. (2011) Prediction of GABA(A) receptor proteins using the concept of Chou's pseudo-amino acid composition and support vector machine. *J. Theor. Biol.*, **281**, 18-23.
125. Mohammad Beigi, M., Behjati, M. and Mohabatkar, H. (2011) Prediction of metalloproteinase family based on the concept of Chou's pseudo amino acid composition using a machine learning approach. *Journal of Structural and Functional Genomics*, **12**, 191-197.
126. Qiu, J.D., Sun, X.Y., Suo, S.B., Shi, S.P., Huang, S.Y., Liang, R.P. and Zhang, L. (2011) Predicting homo-oligomers and hetero-oligomers by pseudo-amino acid composition: an approach from discrete wavelet transformation. *Biochimie*, **93**, 1132-1138.
127. Qiu, J.D., Suo, S.B., Sun, X.Y., Shi, S.P. and Liang, R.P. (2011) OligoPred: A web-server for predicting homo-oligomeric proteins by incorporating discrete wavelet transform into Chou's pseudo amino acid composition. *Journal of Molecular Graphics & Modelling*, **30**, 129-134.
128. Shi, R. and Xu, C. (2011) Prediction of rat protein subcellular localization with pseudo amino Acid composition based on multiple sequential features. *Protein and Peptide Letters*, **18**, 625-633.
129. Shu, M., Cheng, X., Zhang, Y., Wang, Y., Lin, Y., Wang, L. and Lin, Z. (2011) Predicting the Activity of ACE Inhibitory Peptides with a Novel Mode of Pseudo Amino Acid Composition. *Protein & Peptide Letters*, **18**, 1233-1243.
130. Wang, D., Yang, L., Fu, Z. and Xia, J. (2011) Prediction of thermophilic protein with pseudo amino Acid composition: an approach from combined feature selection and reduction. *Protein & Peptide Letters*, **18**, 684-689.
131. Wang, W., Geng, X.B., Dou, Y., Liu, T. and Zheng, X. (2011) Predicting protein

subcellular localization by pseudo amino Acid composition with a segment-weighted and features-combined approach. *Protein and Peptide Letters*, **18**, 480-487.

132. Xiao, X. and Chou, K.C. (2011) Using pseudo amino acid composition to predict protein attributes via cellular automata and other approaches. *Current Bioinformatics*, **6**, 251-260.
133. Xiao, X., Wang, P. and Chou, K.C. (2011) GPCR-2L: Predicting G protein-coupled receptors and their types by hybridizing two different modes of pseudo amino acid compositions. *Molecular Biosystems*, **7**, 911-919.
134. Zhou, X., Li, Z., Dai, Z. and Zou, X. (2011) Predicting methylation status of human DNA sequences by pseudo-trinucleotide composition. *Talanta*, **85**, 1143-1147.
135. Zia Ur, R. and Khan, A. (2011) Prediction of GPCRs with Pseudo Amino Acid Composition: Employing Composite Features and Grey Incidence Degree Based Classification. *Protein & Peptide Letters*, **18**, 872-878.
136. Zou, D., He, Z., He, J. and Xia, Y. (2011) Supersecondary structure prediction using Chou's pseudo amino acid composition. *J. Comput. Chem.*, **32**, 271-278.
137. Cao, J.Z., Liu, W.Q. and Gu, H. (2012) Predicting Viral Protein Subcellular Localization with Chou's Pseudo Amino Acid Composition and Imbalance-Weighted Multi-Label K-Nearest Neighbor Algorithm. *Protein and Peptide Letters*, **19**, 1163-1169.
138. Chen, C., Shen, Z.B. and Zou, X.Y. (2012) Dual-Layer Wavelet SVM for Predicting Protein Structural Class Via the General Form of Chou's Pseudo Amino Acid Composition. *Protein & Peptide Letters*, **19**, 422-429.
139. Chen, Y.L., Li, Q.Z. and Zhang, L.Q. (2012) Using increment of diversity to predict mitochondrial proteins of malaria parasite: integrating pseudo-amino acid composition and structural alphabet. *Amino Acids*, **42**, 1309-1316.
140. Fan, G.L. and Li, Q.Z. (2012) Predict mycobacterial proteins subcellular locations by incorporating pseudo-average chemical shift into the general form of Chou's pseudo amino acid composition. *J. Theor. Biol.*, **304**, 88-95.
141. Fan, G.L. and Li, Q.Z. (2012) Predicting protein submitochondria locations by combining different descriptors into the general form of Chou's pseudo amino acid composition. *Amino Acids*, **43**, 545-555.
142. Gao, Q.B., Zhao, H., Ye, X. and He, J. (2012) Prediction of pattern recognition receptor family using pseudo-amino acid composition. *Biochem. Biophys. Res. Commun.*, **417**, 73-77.
143. Li, L.Q., Zhang, Y., Zou, L.Y., Zhou, Y. and Zheng, X.Q. (2012) Prediction of Protein Subcellular Multi-Localization Based on the General form of Chou's Pseudo Amino Acid Composition. *Protein & Peptide Letters*, **19**, 375-387.
144. Lin, W.Z., Fang, J.A., Xiao, X. and Chou, K.C. (2012) Predicting Secretory Proteins of Malaria Parasite by Incorporating Sequence Evolution Information into Pseudo Amino Acid Composition via Grey System Model. *PLoS One*, **7**, e49040.
145. Liu, L., Hu, X.Z., Liu, X.X., Wang, Y. and Li, S.B. (2012) Predicting Protein Fold Types by the General Form of Chou's Pseudo Amino Acid Composition: Approached from Optimal Feature Extractions. *Protein & Peptide Letters*, **19**,

439-449.
146. Nanni, L., Brahnam, S. and Lumini, A. (2012) Wavelet images and Chou's pseudo amino acid composition for protein classification. *Amino Acids*, **43**, 657-665.
147. Nanni, L., Lumini, A., Gupta, D. and Garg, A. (2012) Identifying bacterial virulent proteins by fusing a set of classifiers based on variants of Chou's pseudo amino acid composition and on evolutionary information. *IEEE/ACM Trans Comput Biol Bioinform*, **9**, 467-475.
148. Niu, X.H., Hu, X.H., Shi, F. and Xia, J.B. (2012) Predicting Protein Solubility by the General Form of Chou's Pseudo Amino Acid Composition: Approached from Chaos Game Representation and Fractal Dimension. *Protein & Peptide Letters*, **19**, 940-948.
149. Ren, L.Y., Zhang, Y.S. and Gutman, I. (2012) Predicting the Classification of Transcription Factors by Incorporating their Binding Site Properties into a Novel Mode of Chou's Pseudo Amino Acid Composition. *Protein & Peptide Letters*, **19**, 1170-1176.
150. Thomsen, M.C. and Nielsen, M. (2012) Seq2Logo: a method for construction and visualization of amino acid binding motifs and sequence profiles including sequence weighting, pseudo counts and two-sided representation of amino acid enrichment and depletion. *Nucleic Acids Res.*, **40**, W281-287.
151. Wang, J., Li, Y., Wang, Q., You, X., Man, J., Wang, C. and Gao, X. (2012) ProClusEnsem: predicting membrane protein types by fusing different modes of pseudo amino acid composition. *Comput Biol Med*, **42**, 564-574.
152. Yu, X., Zheng, X., Liu, T., Dou, Y. and Wang, J. (2012) Predicting subcellular location of apoptosis proteins with pseudo amino acid composition: approach from amino acid substitution matrix and auto covariance transformation. *Amino Acids*, **42**, 1619-1625.
153. Zhao, X.W., Ma, Z.Q. and Yin, M.H. (2012) Predicting protein-protein interactions by combing various sequence- derived features into the general form of Chou's Pseudo amino acid composition. *Protein & Peptide Letters*, **19**, 492-500.
154. Zia Ur, R. and Khan, A. (2012) Identifying GPCRs and their Types with Chou's Pseudo Amino Acid Composition: An Approach from Multi-scale Energy Representation and Position Specific Scoring Matrix. *Protein & Peptide Letters*, **19**, 890-903.
155. Chen, W., Feng, P.M., Lin, H. and Chou, K.C. (2013) iRSpot-PseDNC: identify recombination spots with pseudo dinucleotide composition *Nucleic Acids Res.*, **41**, e68.
156. Chen, Y.K. and Li, K.B. (2013) Predicting membrane protein types by incorporating protein topology, domains, signal peptides, and physicochemical properties into the general form of Chou's pseudo amino acid composition. *J. Theor. Biol.*, **318**, 1-12.
157. Fan, G.L. and Li, Q.Z. (2013) Discriminating bioluminescent proteins by incorporating average chemical shift and evolutionary information into the general form of Chou's pseudo amino acid composition. *J. Theor. Biol.*, **334**, 45-51.

158. Feng, P.M., Chen, W., Lin, H. and Chou, K.C. (2013) iHSP-PseRAAAC: Identifying the heat shock protein families using pseudo reduced amino acid alphabet composition. *Anal. Biochem.*, **442**, 118-125.
159. Georgiou, D.N., Karakasidis, T.E. and Megaritis, A.C. (2013) A short survey on genetic sequences, Chou's pseudo amino acid composition and its combination with fuzzy set theory. *The Open Bioinformatics Journal*, **7**, 41-48.
160. Gupta, M.K., Niyogi, R. and Misra, M. (2013) An alignment-free method to find similarity among protein sequences via the general form of Chou's pseudo amino acid composition. *SAR QSAR Environ Res (SAR AND QSAR IN ENVIRONMENTAL RESEARCH)*, **24**, 597-609.
161. Huang, C. and Yuan, J. (2013) Using radial basis function on the general form of Chou's pseudo amino acid composition and PSSM to predict subcellular locations of proteins with both single and multiple sites. *Biosystems*, **113**, 50-57.
162. Huang, C. and Yuan, J.Q. (2013) A Multilabel Model Based on Chou's Pseudo-Amino Acid Composition for Identifying Membrane Proteins with Both Single and Multiple Functional Types. *J. Membr. Biol.*, **246**, 327-334.
163. Huang, C. and Yuan, J.Q. (2013) Predicting protein subchloroplast locations with both single and multiple sites via three different modes of Chou's pseudo amino acid compositions. *J. Theor. Biol.*, **335**, 205-212.
164. Khosravian, M., Faramarzi, F.K., Beigi, M.M., Behbahani, M. and Mohabatkar, H. (2013) Predicting Antibacterial Peptides by the Concept of Chou's Pseudo-amino Acid Composition and Machine Learning Methods. *Protein & Peptide Letters*, **20**, 180-186.
165. Lin, H., Ding, C., Yuan, L.F., Chen, W., Ding, H., Li, Z.Q., Guo, F.B., Hung, J. and Rao, N.N. (2013) Predicting subchloroplast locations of proteins based on the general form of Chou's pseudo amino acid composition: approached from optimal tripeptide composition. *International Journal of Biomathematics*, **6**, Article Number: 1350003.
166. Liu, B., Wang, X., Zou, Q., Dong, Q. and Chen, Q. (2013) Protein remote homology detection by combining Chou's pseudo amino acid composition and profile-based protein representation. *Molecular Informatics*, **32**, 775-782.
167. Mohabatkar, H., Beigi, M.M., Abdolahi, K. and Mohsenzadeh, S. (2013) Prediction of Allergenic Proteins by Means of the Concept of Chou's Pseudo Amino Acid Composition and a Machine Learning Approach. *Medicinal Chemistry*, **9**, 133-137.
168. Qin, Y.F., Zheng, L. and Huang, J. (2013) Locating apoptosis proteins by incorporating the signal peptide cleavage sites into the general form of Chou's Pseudo amino acid composition. *Int. J. Quantum Chem.*, **113**, 1660-1667.
169. Sarangi, A.N., Lohani, M. and Aggarwal, R. (2013) Prediction of Essential Proteins in Prokaryotes by Incorporating Various Physico-chemical Features into the General form of Chou's Pseudo Amino Acid Composition. *Protein Pept Lett*, **20**, 781-795.
170. Wan, S., Mak, M.W. and Kung, S.Y. (2013) GOASVM: A subcellular location predictor by incorporating term-frequency gene ontology into the general form of Chou's pseudo-amino acid composition. *J. Theor. Biol.*, **323**, 40-48.

171. Wang, X., Li, G.Z. and Lu, W.C. (2013) Virus-ECC-mPLoc: a multi-label predictor for predicting the subcellular localization of virus proteins with both single and multiple sites based on a general form of Chou's pseudo amino acid composition. *Protein & Peptide Letters*, **20**, 309-317.
172. Xiaohui, N., Nana, L., Jingbo, X., Dingyan, C., Yuehua, P., Yang, X., Weiquan, W., Dongming, W. and Zengzhen, W. (2013) Using the concept of Chou's pseudo amino acid composition to predict protein solubility: An approach with entropies in information theory. *J. Theor. Biol.*, **332C**, 211-217.
173. Zhou, X., Li, Z., Dai, Z. and Zou, X. (2013) Predicting promoters by pseudo-trinucleotide compositions based on discrete wavelets transform. *J. Theor. Biol.*, **319**, 1-7.
174. Chen, W., Feng, P.M., Deng, E.Z., Lin, H. and Chou, K.C. (2014) iTIS-PseTNC: a sequence-based predictor for identifying translation initiation site in human genes using pseudo trinucleotide composition. *Anal. Biochem.*, **462**, 76-83.
175. Chen, W., Feng, P.M., Lin, H. and Chou, K.C. (2014) iSS-PseDNC: identifying splicing sites using pseudo dinucleotide composition. *Biomed Research International (BMRI)*, **2014**, 623149.
176. Chen, W., Lei, T.Y., Jin, D.C., Lin, H. and Chou, K.C. (2014) PseKNC: a flexible web-server for generating pseudo K-tuple nucleotide composition. *Anal. Biochem.*, **456**, 53-60.
177. Feng, P., Jiang, N. and Liu, N. (2014) Prediction of DNase I hypersensitive sites by using pseudo nucleotide compositions. *The Scientific World Journal*, **2014**, 740506.
178. Hajisharifi, Z., Piryaiee, M., Mohammad Beigi, M., Behbahani, M. and Mohabatkar, H. (2014) Predicting anticancer peptides with Chou's pseudo amino acid composition and investigating their mutagenicity via Ames test. *J. Theor. Biol.*, **341**, 34-40.
179. Jia, C., Lin, X. and Wang, Z. (2014) Prediction of Protein S-Nitrosylation Sites Based on Adapted Normal Distribution Bi-Profile Bayes and Chou's Pseudo Amino Acid Composition. *Int J Mol Sci*, **15**, 10410-10423.
180. Kong, L., Zhang, L. and Lv, J. (2014) Accurate prediction of protein structural classes by incorporating predicted secondary structure information into the general form of Chou's pseudo amino acid composition. *J. Theor. Biol.*, **344**, 12-18.
181. Liu, B., Xu, J., Lan, X., Xu, R., Zhou, J., Wang, X. and Chou, K.C. (2014) iDNA-Prot|dis: identifying DNA-binding proteins by incorporating amino acid distance-pairs and reduced alphabet profile into the general pseudo amino acid composition. *PLoS ONE*, **9**, e106691.
182. Mondal, S. and Pai, P.P. (2014) Chou's pseudo amino acid composition improves sequence-based antifreeze protein prediction. *J. Theor. Biol.*, **356**, 30-35.
183. Nanni, L., Brahnam, S. and Lumini, A. (2014) Prediction of protein structure classes by incorporating different protein descriptors into general Chou's pseudo amino acid composition. *J. Theor. Biol.*, **360C**, 109-116.
184. Xu, R., Zhou, J., Liu, B., He, Y.A., Zou, Q., Wang, X. and Chou, K.C. (2014) Identification of DNA-binding proteins by incorporating evolutionary

information into pseudo amino acid composition via the top-n-gram approach. *Journal of Biomolecular Structure & Dynamics (JBSD)*, **doi: 10.1080/07391102.2014.968624**.

185. Xu, Y., Wen, X., Wen, L.S., Wu, L.Y., Deng, N.Y. and Chou, K.C. (2014) iNitro-Tyr: Prediction of nitrotyrosine sites in proteins with general pseudo amino acid composition. *PLoS ONE*, **9**, e105018.
186. Zhang, J., Sun, P., Zhao, X. and Ma, Z. (2014) PECM: Prediction of extracellular matrix proteins using the concept of Chou's pseudo amino acid composition. *J. Theor. Biol.*, **363**, 412-418.
187. Zhang, L., Zhao, X. and Kong, L. (2014) Predict protein structural class for low-similarity sequences by evolutionary difference information into the general form of Chou's pseudo amino acid composition. *J. Theor. Biol.*, **355**, 105-110.
188. Zuo, Y.C., Peng, Y., Liu, L., Chen, W., Yang, L. and Fan, G.L. (2014) Predicting peroxidase subcellular location by hybridizing different descriptors of Chou's pseudo amino acid patterns. *Anal. Biochem.*, **458**, 14-19.
189. Huang, C. and Yuan, J.Q. (2015) Simultaneously Identify Three different Attributes of Proteins by Fusing their Three different Modes of Chou's Pseudo Amino Acid Compositions. *Protein Pept Lett*.
190. Khan, Z.U., Hayat, M. and Khan, M.A. (2015) Discrimination of acidic and alkaline enzyme using Chou's pseudo amino acid composition in conjunction with probabilistic neural network model. *J. Theor. Biol.*, **365**, 197-203.
191. Kumar, R., Srivastava, A., Kumari, B. and Kumar, M. (2015) Prediction of beta-lactamase and its class by Chou's pseudo-amino acid composition and support vector machine. *J. Theor. Biol.*, **365**, 96-103.
192. Liu, B., Fang, L., Liu, F., Wang, X., Chen, J. and Chou, K.C. (2015) Identification of real microRNA precursors with a pseudo structure status composition approach. *PLoS ONE*, **10**, e0121501.
193. Liu, B., Fang, L., Liu, F., Wang, X. and Chou, K.C. (2015) iMiRNA-PseDPC: microRNA precursor identification with a pseudo distance-pair composition approach. *Journal of Biomolecular Structure & Dynamics (JBSD)*, http://dx.doi.org/10.1080/07391102.07392015.01014422.
194. Liu, Z., Xiao, X., Qiu, W.R. and Chou, K.C. (2015) iDNA-Methyl: Identifying DNA methylation sites via pseudo trinucleotide composition. *Anal. Biochem.*, **474**, 69-77.
195. Chen, W., Lin, H., Feng, P.M., Ding, C., Zuo, Y.C. and Chou, K.C. (2012) iNuc-PhysChem: A Sequence-Based Predictor for Identifying Nucleosomes via Physicochemical Properties. *PLoS ONE*, **7**, e47843.
196. Feng, P., Chen, W. and Lin, H. (2014) Prediction of CpG island methylation status by integrating DNA physicochemical properties. *Genomics*, **104**, 229-233.
197. Qiu, W.R., Xiao, X., Lin, W.Z. and Chou, K.C. (2014) iUbiq-Lys: Prediction of lysine ubiquitination sites in proteins by extracting sequence evolution information via a grey system model *Journal of Biomolecular Structure and Dynamics (JBSD)* **doi:10.1080/07391102.2014.968875**.
198. Min, J.L., Xiao, X. and Chou, K.C. (2013) iEzy-Drug: A web server for identifying the interaction between enzymes and drugs in cellular networking. *BioMed*

Research International (BMRI), **2013**, 701317.
199. Xiao, X., Min, J.L., Wang, P. and Chou, K.C. (2013) iGPCR-Drug: A web server for predicting interaction between GPCRs and drugs in cellular networking. *PLoS ONE*, **8**, e72234.
200. Xiao, X., Min, J.L., Wang, P. and Chou, K.C. (2013) Predict drug-protein interaction in cellular networking. *Current Topics in Medicinal Chemistry*, **13**, 1707-1712.
201. Fan, Y.N., Xiao, X., Min, J.L. and Chou, K.C. (2014) iNR-Drug: Predicting the interaction of drugs with nuclear receptors in cellular networking. *Intenational Journal of Molecular Sciences*, **15**, 4915-4937.
202. Xiao, X., Min, J.L., Lin, W.Z., Liu, Z., Cheng, X. and Chou, K.C. (2014) iDrug-Target: predicting the interactions between drug compounds and target proteins in cellular networking via the benchmark dataset optimization approach. *Journal of Biomolecular Structure & Dynamics (JBSD)* doi: 10.1080/07391102.07392014.07998710.
